# A Unified Framework for the Infection Dynamics of Zoonotic Spillover and Spread

**DOI:** 10.1371/journal.pntd.0004957

**Published:** 2016-09-02

**Authors:** Giovanni Lo Iacono, Andrew A. Cunningham, Elisabeth Fichet-Calvet, Robert F. Garry, Donald S. Grant, Melissa Leach, Lina M. Moses, Gordon Nichols, John S. Schieffelin, Jeffrey G. Shaffer, Colleen T. Webb, James L. N. Wood

**Affiliations:** 1 Department of Veterinary Medicine, Disease Dynamics Unit, University of Cambridge, Cambridge, United Kingdom; 2 Environmental Change Department, Centre for Radiation, Chemical and Environmental Hazards, Public Health England, London, United Kingdom; 3 Institute of Zoology, Zoological Society of London, United Kingdom; 4 Bernhard-Nocht Institute of Tropical Medicine, Hamburg, Germany; 5 Department of Microbiology and Immunology, Tulane University, New Orleans, Louisiana, United States of America; 6 Lassa Fever Program, Kenema Government Hospital, Kenema, Sierra Leone; 7 Institute of Development Studies, University of Sussex, Brighton, United Kingdom; 8 Gastrointestinal, Emerging and Zoonotic Infections, Public Health England, London, United Kingdom; 9 Sections of Infectious Disease, Departments of Pediatrics and Internal Medicine, School of Medicine, Tulane University, New Orleans, Louisiana, United States of America; 10 Department of Biostatistics and Bioinformatics, Tulane School of Public Health and Tropical Medicine, New Orleans, Louisiana, United States of America; 11 Department of Biology, Colorado State University, Fort Collins, Colorado, United States of America; University of California Davis, UNITED STATES

## Abstract

A considerable amount of disease is transmitted from animals to humans and many of these zoonoses are neglected tropical diseases. As outbreaks of SARS, avian influenza and Ebola have demonstrated, however, zoonotic diseases are serious threats to global public health and are not just problems confined to remote regions. There are two fundamental, and poorly studied, stages of zoonotic disease emergence: ‘spillover’, *i.e.* transmission of pathogens from animals to humans, and ‘stuttering transmission’, *i.e.* when limited human-to-human infections occur, leading to self-limiting chains of transmission. We developed a transparent, theoretical framework, based on a generalization of Poisson processes with memory of past human infections, that unifies these stages. Once we have quantified pathogen dynamics in the reservoir, with some knowledge of the mechanism of contact, the approach provides a tool to estimate the likelihood of spillover events. Comparisons with independent agent-based models demonstrates the ability of the framework to correctly estimate the relative contributions of human-to-human vs animal transmission. As an illustrative example, we applied our model to Lassa fever, a rodent-borne, viral haemorrhagic disease common in West Africa, for which data on human outbreaks were available. The approach developed here is general and applicable to a range of zoonoses. This kind of methodology is of crucial importance for the scientific, medical and public health communities working at the interface between animal and human diseases to assess the risk associated with the disease and to plan intervention and appropriate control measures. The Lassa case study revealed important knowledge gaps, and opportunities, arising from limited knowledge of the temporal patterns in reporting, abundance of and infection prevalence in, the host reservoir.

## Author Summary

Many dangerous diseases emerge via spillover from animals, with limited human-to-human infection (stuttering-transmission) often being the first stage of human disease spread. Understanding the conditions (biological, environmental and socio-economic factors) that regulate spillover and disease spread is key to its mitigation. Here we are interested in questions such as: If we have quantified pathogen dynamics in the reservoir, with some knowledge of the mechanism of contact, can we estimate the likelihood of spillover events? Can we tease apart how much the disease is transmitted by animals and how much by humans? We developed a unified mathematical framework, based on Poisson processes with memory of past events, to understand the dynamics of spillover and stuttering-transmission. This framework, which can be applied across the disease transmission spectrum, allows the teasing apart of the disease burden attributed to animal-human and human-human transmission. Using this model, we can infer human disease risk based on knowledge of infection patterns in the animal reservoir host and the contact mechanisms required for transmission to humans.

## Introduction

An important class of pathogens are those transmitted from animals to humans (zoonosis). The dangers associated with zoonotic pathogens are twofold. Firstly, the pathogen can adapt to the new human host and acquire the ability to transmit sustainably from human-to-human without the need for continued seeding from the animal reservoir. The pathogens involved occasionally transmit rapidly amongst its immunologically naïve new host causing devastating health impacts as demonstrated by the global SARS outbreak, the swine influenza pandemic and the recent Ebola epidemic, which probably originated from one zoonotic spillover event. Perhaps, however, HIV-1 is the most spectacular case of a recent zoonotic emergence, originating from an endemic infection of chimpanzees in Central Africa. Zoonotic infections are the origin of the majority of established human pathogens [1] of which influenza, measles, smallpox and diphtheria are examples [2]. Secondly, zoonotic pathogens can spill over from animal reservoirs continually and cause a heavy burden of disease. Human rabies from domestic dogs is an important and preventable example.

The origins of major human infectious diseases can be conceptualised as a continuous transition across different epidemiologic stages [3]. The first stage is when a pathogen exclusively infects animals (‘reservoir dynamics’). The second is when the pathogen occasionally jumps to the dead-end-host human population (‘spillover’). This is followed by a third stage, when human-to-human transmission becomes possible but leads only to self-limiting chains of transmission (‘stuttering transmission’). The final stage is when a pathogen gains the ability to transmit effectively between humans and no longer requires zoonotic transmission [3]. An additional scenario is when the pathogen infects both animals and humans in a sustainable manner.

Measuring and predicting cross-species transmission is extremely difficult. This is because spillovers are often, but not always (as the situation for Lassa Fever demonstrates), rare events driven by the complex interactions of multiple causes, including ecological factors (*e.g.* presence of hosts with differing degrees of susceptibility and periodicity in their abundance), epidemiological and genetic factors (*e.g.* a broad set of pathogen life histories and periodicity of infection prevalence), and anthropogenic activities (*e.g.* land-use and behavioural changes affecting direct and indirect interactions with reservoir hosts) [4]. Particularly challenging are zoonoses with stuttering transmission, as separating the contribution of animal-to-human from human-to-human transmission is extremely difficult.

Not surprisingly, theoretical [5–7] and experimental studies able to disentangle the many complex aspects of transmission at the animal-human interface are scarce [3, 8]. An increasing body of research recognises the need for a new paradigm integrating biological, social and environmental sciences with mathematical modelling to explain the mechanisms and impacts of zoonotic emergence [9, 10].

Understanding zoonotic spillover and stuttering transmission are, therefore, two very important public health challenges. The scientific, public health and medical communities working at the interface of animal and human pathogens are challenged with many questions, such as: i) if we know the pathogen abundance and prevalence in the reservoir and have some knowledge of the mechanism of contact, can we estimate the likelihood of the next spillover event? ii) is there a signature in the patterns of disease occurrence that enables us to distinguish the spillover (animal-to-human) burden from the stuttering chain (human-to-human) burden? iii) how is zoonotic risk driven by specific social, economic, environmental and biological factors?

Mathematical modelling has been used before to estimate the relative contributions of zoonotic spillover and human-to-human transmission, [11]. This approach was based on rarely available information of nosocomial and extra-nosocomial outbreaks that were known to be instances of pure human-to-human chains. More general methods are needed. Here, we developed a unified mathematical framework for spillover and stuttering chain processes. These are conceptually similar mechanisms; they are both arrival processes. The key difference is that zoonotic spillovers are assumed to arise from random and independent contacts with the reservoir with no influence of past infections (assuming no depletion of susceptibles, *i.e.* the pool of people who can be infected by contact with the reservoir or humans). In contrast, a stuttering chain, which arises from human-to-human transmission, is affected by the number of past human infections as each infected person can also trigger a chain of new cases. Zoonotic spillovers are also affected by past events when depletion of susceptibles, through death or development of sterilising immunity, is important.

**Fig 1:**
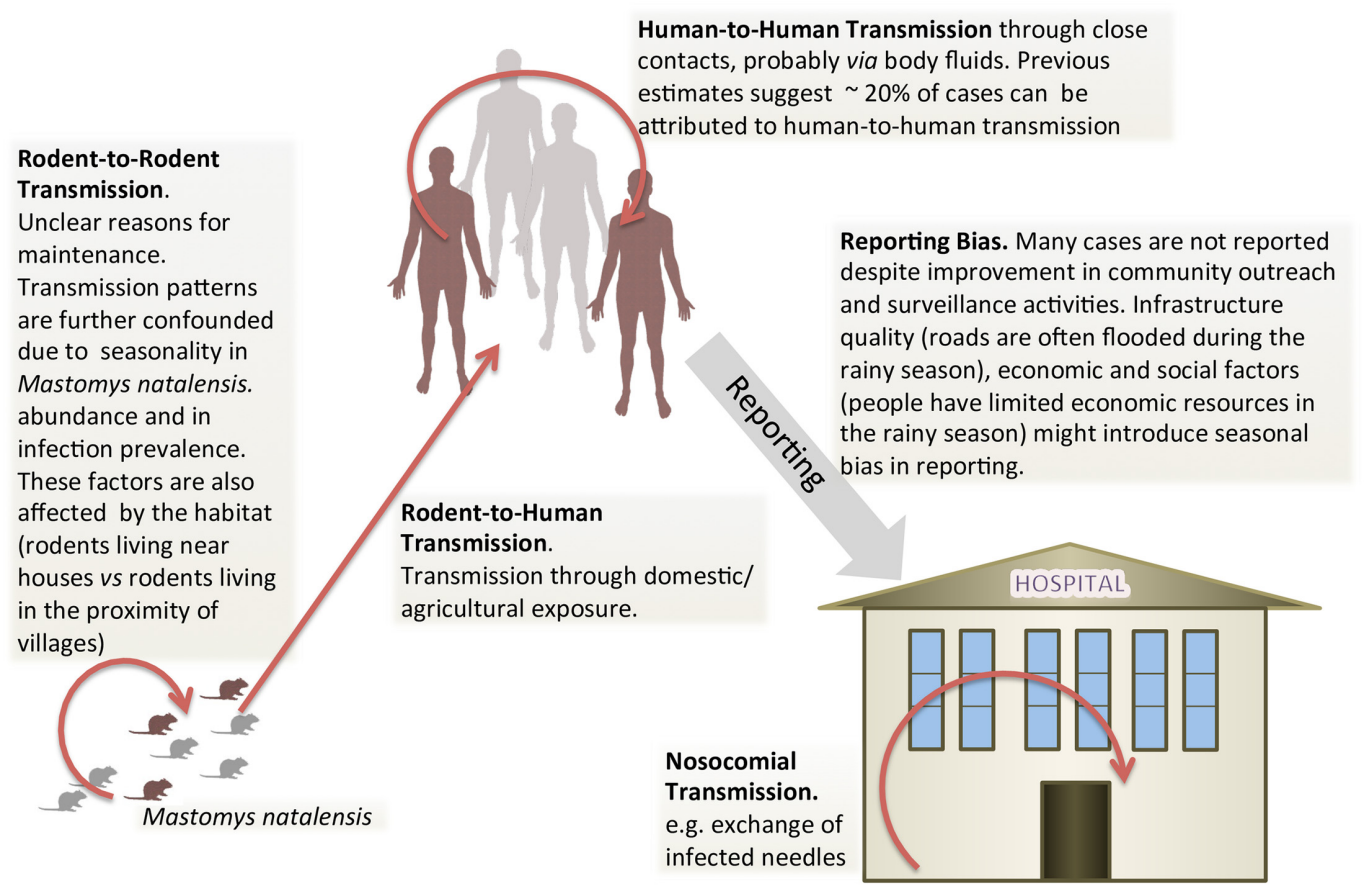
Schematic of the transmission cycle of Lassa virus in *Mastomys natalensis* and in humans.

Mathematically, zoonotic spillovers are described by Poisson processes (Cox processes if stochasticity in the rate of infection becomes important) or by self-correcting (*i.e.* decreasing rate of infection) processes if depletion of susceptibles occurs, while stuttering chains are described by a combination of self-exciting (*i.e.* increasing rate of infection), due to previous human infections, and self-correcting due to depletion of susceptibles, processes (see Table S1 in S2 Text). We tested different models by comparing their predictions with the corresponding outputs from independently-simulated epidemics generated by an agent based model (ABM). As an illustrative example, we also applied the final model to Lassa Fever (LF), a zoonotic, viral haemorrhagic disease common in West Africa, for which data from Kenema Government Hospital (KGH) in Sierra Leone [12] are available. LF represents an important model for this kind of study (Fig 1). The disease reservoir is *Mastomys natalensis* [13], one of the most common African rodents, but an important proportion of the burden of disease is ascribable to human-to-human transmission; this is supported by the arguments presented in [11] and by a recent case of secondary transmission of locally acquired Lassa fever in Cologne, Germany [14]. This case study was particularly instructive, revealing important challenges in current knowledge of LF, thus informing the direction of future research.

## Materials and Methods

The proposed method is general but for illustrative purposes the presentation is based on the LF system exemplified in Fig 1. For the LF case study, we used data abstracted from patient medical records and LF diagnostic tests for 1002 suspected LF cases who presented to the KGH Lassa Ward from 27^th^ of April 2010 to the 31^st^ of January 2012 [11, 12]. For the list of symbols see also Supporting Information, S1 Text.

### The distribution of spillover events

The phenomenology of spillover events ought to be linked with disease dynamics in the reservoir and the mechanism of contact between species. We assume that LF is caused by independent random ‘contacts’ (mediated by contaminated food, fomites etc.) between humans and rodents. Thus the probability *P* that *k* events occur during a time *τ* (*e.g.* number of admissions to hospital in one week) can be described by a stochastic Poisson process:

$$P\left(k\right)=\frac{\exp^{-\lambda \tau }{\left(\lambda \tau \right)}^{k}}{k!}$$ (1)

where *λ* is a parameter (*rate*) representing the expected number of zoonotic spillovers per time unit. The parameter *λ* is expected to depend on other drivers [15]. In the simplest scenario the human population is uniformly subjected to random and independent contacts with the reservoir. Only a fraction of these contacts, equal to the infection prevalence of the reservoir, are a potential source of infection. Accordingly, we assume:

$$\lambda =N_{H}Pr_{R}\left(N_{R}\right)\chi_{R}\eta_{R}\left(N_{R}\right)$$ (2)

where *N*_*H*_ is the human population size, *i.e.* the total number of people in a suitable area A, *e.g.* a village; *Pr*_*R*_(*N*_*R*_) is the prevalence of infected rodents; *χ*_*R*_ is a parameter combining two complex mechanisms: the ability of the reservoir to excrete a suitable dosage of the virus and the human response to it. We refer to this parameter as infection-response efficiency, and we formally define it as the product of the probability that the virus is excreted from the reservoir and the probability that humans acquire infection when challenged with the virus. *η*_*R*_(*N*_*R*_) is a measure of exposure, given by the product $\eta_{R}\left(N_{R}\right)=\xi \left(N_{R}\right)A$ where *ξ*(*N*_*R*_) is the probability of a contact (direct or mediated) between *a single* member of the human population and the population of *N*_*R*_ rodents per time unit and area unit. Both the pathogen prevalence, *Pr*_*R*_, and the exposure, *η*_*R*_, are expected to be functions of rodent abundance, *N*_*R*_, although a clear evidence of correlation between LASV prevalence and *M. nataliensis* abundance is lacking. The area A essentially depends on the dispersal range of the rodents and, in the presence of human-to-human transmission, on the mobility of people. Here we assumed that the area A used is suitable for considering the system closed (no change in the population apart from the disease induced mortality) and for assuming uniform mixing, *i.e.* each person is equally in contact with each other and with the rodent population. As in the current model the size A of the system is fixed, we consider the overall parameter *η*_*R*_(*N*_*R*_). Here and throughout, we refer to the quantities *N*_*H*_, *Pr*_*R*_(*N*_*R*_), *χ*_*R*_, *η*_*R*_(*N*_*R*_) (and also *χ*_*N*_ and *η*_*R*_(*N*_*H*_) defined below) as constituent factors.

The assumption that the system is closed can be relaxed. The simplest approach would be capturing the phenomenology of births, deaths and migrations by allowing a time-dependent functional form for the human population size *N*_*H*_ = *N*_*H*_(*t*). Alternatively, changes in the human population size can arise from implementing an appropriate population dynamics model for *N*_*H*_. The approach can be further extended to incorporate explicitly-spatial effects by building an interconnected meta-population model based on homogeneous regions and allowing immigration/emigration of individuals.

Quantities such as the rodent population size, *N*_*R*_, and infection prevalence, *Pr*, are often seasonal therefore the rate *λ* ought to be explicitly time-dependent resulting in a non-homogeneous Poisson process. Most importantly, all the terms in Eq (2), *i.e.* rodent population, *N*_*R*_, infection prevalence, *Pr*, human population size, *N*_*H*_, and the infection-response efficiency, *χ*_*R*_, are in general, stochastic. Thus the parameter *λ* in Eq (1) should be replaced with a random variable leading to the so-called doubly stochastic or Cox process. When the rate *λ* is a gamma-distributed variable, the Cox process is described by a negative binomial distribution (S3 Text). After some algebra based on well-known properties of the negative binomial distribution, we can present further relationships between some parameters of the negative binomial distribution (including mean *μ* and variance *σ*^2^ that uniquely determine the distribution) and the mean *μ*_*λ*_ and variance $\sigma_{\lambda }^{2}$ of the associated gamma-distribution for the rate *λ* (*i.e.* *μ* = *μ*_*λ*_, $\sigma^{2}=\sigma_{\lambda }^{2}+\mu_{\lambda }$, see Table S1 in S3 Text).

As is known, when $\sigma_{\lambda }^{2}$ approaches zero, then the negative binomial approaches a standard Poisson distribution. The properties shown in Table S1 in S3 Text, however, have important implications for quantifying the risk of spillovers. To estimate the probability of a spillover, it is sufficient to know the value of the parameters *μ* and *σ*^2^. These, in turn, can be estimated from the mean and variance, *μ*_*λ*_ and $\sigma_{\lambda }^{2}$, in the rate *λ*, which, ultimately depend on the constituent factors.

### Inferring the parameters *μ*_*λ*_ and $\sigma_{\lambda }^{2}$ from the drivers of transmission

Based on the hypothesis posed in Eq (2), we show how to infer the mean and variances *μ*_*λ*_ and $\sigma_{\lambda }^{2}$ directly from knowledge of the human population size, *N*_*H*_, the abundance of rodents, *N*_*R*_, and also the infection-response efficiency, *χ*_*R*_. Since we expect that *N*_*R*_, *N*_*H*_ and *χ*_*R*_ are independent random variables, the mean value of the rate *λ* is given by the product $\mu_{\lambda }\;=\;\mu_{N_{H}}\;\mu_{\eta_{R}}Pr_{R}\;\mu_{\chi_{R}}$, where $\mu_{N_{H}}$ and $\mu_{\chi_{R}}$ are the mean values associated with the size of the human population, *N*_*H*_, and the infection-response efficiency, *χ*_*R*_; $\mu_{\eta_{R}}Pr_{R}$ is the mean value of the random variable arising from the product *η*_*R*_(*N*_*R*_)*Pr*_*R*_(*N*_*R*_), *i.e.* the exposure to the infected reservoir only (while *η*_*R*_(*N*_*R*_) is the ‘exposure to the reservoir’, irrespective of this being infected or not). Similarly, the variance $\sigma_{\lambda }^{2}$ can be estimated as

$$\sigma_{\lambda }^{2}\approx {\left[\eta_{R}\left(N_{R}\right)Pr_{R}\left(N_{R}\right)\chi_{R}\right]}^{2}\sigma_{N_{H}}^{2}+{\left[N_{H}\chi_{R}\frac{\partial \sigma \left(N_{R}\right)Pr_{R}\left(N_{R}\right)}{\partial N_{R}}\right]}^{2}\sigma_{N_{R}}^{2}+{\left[N_{H}\eta_{R}\left(N_{R}\right)Pr_{R}\left(N_{R}\right)\right]}^{2}\sigma_{\chi_{R}}^{2}$$ (3)

where we used the usual approximation:

$$\sigma_{f}^{2}\approx {\left|\frac{\partial f}{\partial X}\right|}^{2}\sigma_{X}^{2}+{\left|\frac{\partial f}{\partial Y}\right|}^{2}\sigma_{Y}^{2}+2\frac{\partial f}{\partial X}\frac{\partial f}{\partial Y}{\text{cov}}_{XY}.$$ (4)

for a function of two random variables *X* and *Y* where *N*_*R*_, *N*_*H*_ and *χ*_*R*_ are independent. Of course, if $\sigma_{N_{H}}^{2}\approx \sigma_{N_{R}}^{2}\approx \sigma_{\chi_{R}}^{2}\approx 0$ then the spillover process is well approximated by a standard Poisson process.

In some situations the explicit dependency of the quantity *η*_*R*_(*N*_*R*_)*Pr*_*R*_(*N*_*R*_) on the abundance of the reservoir is known or can be crudely estimated. Then, the mean and variance *μ*_*λ*_ and $\sigma_{\lambda }^{2}$ can be evaluated directly from the *N*_*R*_ as shown for a range of relevant situations in Table S1 in S4 Text (see also Davis *et al.* [15]).

### Depletion of susceptibles

The model above was derived with the assumption that the number of susceptibles is constant. In a small population, however, the depletion of susceptibles is expected to be an important effect that can result in a self-constraining epidemic. Following model Eq (1), we replaced the (*fixed*) size of the human population *N*_*H*_ with the (*variable*) number of susceptibles, *S*_*H*_. Thus, the probability of observing *k* cases at any time *t*_*j*_ during the interval [(*j* − 1)*τ*, *jτ*] (with *t*_*j*_ ∈ [(*j* − 1)*τ*, *jτ*]) is the piecewise function defined on discrete intervals:

$$\begin{array}{ll} \tilde{P}(k,t_{j}) & =\frac{{\text{exp}}^{-{\tilde{\lambda }}_{j-1}\tau }{\left({\tilde{\lambda }}_{j-1}\tau \right)}^{k}}{k!}\;\;\text{with rate}\\ {\tilde{\lambda }}_{j} & =S_{H}(t_{j})\eta_{R}(N_{R})Pr_{R}(N_{R})\chi_{R} \end{array}$$ (5)

where the time-dependent terms at time *t*_*j*_ are estimated at the end of the previous interval [(*j* − 1)*τ*, *jτ*]. Underlying this choice is the assumption that the time step *τ* is comparable to the transition time from the susceptible to non-susceptible category, and *λ*_*j*_ can be considered constant during this time interval. To estimate *S*_*H*_(*t*_*j*_), we considered the case of an initially susceptible population. For simplicity we assumed no external immigration and that the size of the human population at the initial time is *S*_*H*_(0) = *N*_*H*_. As soon as spillover events start, part of the human population becomes infected; some with resulting life-time immunity and others die. As we consider a closed human population, the number of susceptibles is:

$$S_{H}\left(t_{j}\right)=\left\{\begin{array}{cc} N_{H}-C_{H}\left(t_{j}\right) & \text{if}\;N_{H}>C_{H}\left(t_{j}\right)\\ 0 & \text{otherwise} \end{array}\right.$$ (6)

where *C*_*H*_(*jτ*) represents the cumulative number of people who had been infected at any past time during the interval [0, *jτ*], irrespective of if they recovered or died. This corresponds to:

$$C_{H}\left(t_{j}\right)=C_{H}\left(t_{j-1}\right)+E\left[\tilde{P}\left(k,t_{j}\right)\right]$$ (7)

where $E[\tilde{P}\left(k,t_{j}\right)]$ is the expected number of spillover events during the time-interval [(*j* − 1)*τ*, *jτ*], as $E\left[\tilde{P}\left(k,t_{j}\right)\right]={\tilde{\lambda }}_{j-1}\tau$, thus we have

$$C_{H}\left(t_{j}\right)=C_{H}\left(t_{j-1}\right)+S_{H}\left(t_{j-1}\right)\eta_{R}\left(N_{R}\right)Pr_{R}\left(N_{R}\right)\chi_{R}\tau$$ (8)

The probability $\tilde{P}\left(k,t_{j}\right)$ at time *t*_*j*_ in Eq (5) can be iteratively calculated by replacing the susceptible and cumulative infected, *S*_*H*_ and *C*_*H*_, with their explicit expressions given in Eqs (6) and (8) estimated at the previous time *t*_*j*−1_. Of course, if the depletion of susceptibles is negligible then *S*(*t*_*j*_) ≈ *N*_*H*_ and the model collapses to a standard Poisson process or Cox-process if we allow for stochasticity in the rate. Eq (5) is a particular case of a class of models known Hawkes point processes (see [16] and references therein). We refer to these processes as ‘zoonotic spillover with depletion of susceptibles’ (in mathematical parlance ‘Self-Correcting Poisson’).

### Inclusion of human-to-human transmission: from spillover to stuttering transmission

Hawkes point processes introduced above suggest a natural extension of the current model to include human-to-human transmission. In this context each infection event at time *t*_*j*_, represented by *I*_*H*_(*t*_*j*_), has a certain probability of generating new events. Accordingly, the probability of observing *k* cases at any time *t*_*j*_ ∈ [(*j* − 1)*τ*, *jτ*] is the piecewise function:

$$\begin{array}{lll} \hat{P}(k,t_{j}) & = & \frac{{\text{exp}}^{-{\hat{\lambda }}_{j-1}\tau }{\left({\hat{\lambda }}_{j-1}\tau \right)}^{k}}{k!}\\ {\hat{\lambda }}_{j} & = & \overset{\text{zoonosis}}{\overset{︷}{S_{H}(t_{j})\eta_{R}(N_{R})Pr_{R}(N_{R})\chi_{R}}}+\\ & & \overset{\text{human}-\text{to}-\text{human}}{\overset{︷}{S_{H}(t_{j})\eta_{H}(N_{H})Pr_{H}(N_{H},t_{j})\chi_{H}}}\\ Pr_{H}(N_{H},t_{j}) & = & \frac{I_{H}(t_{j})}{S_{H}(t_{j})+I_{H}(t_{j})+R_{H}(t_{j})} \end{array}$$ (9)

where *η*_*H*_(*N*_*H*_) is the probability that *a single* person is in contact with any other member of the human population per time unit; *χ*_*H*_ is the human analogue of the reservoir infection-response efficiency, *i.e.* the product of the probability that the virus is excreted from a person and the probability that a person acquires infection when exposed to the virus; *Pr*_*H*_(*N*_*H*_) is the infection prevalence in the human population, which is the proportion of infected members *I*_*H*_(*t*_*j*_) in relation to the total size of the current population, *i.e.* for an SIR-type of model *S*_*H*_(*t*_*j*_) + *I*_*H*_(*t*_*j*_) + *R*_*H*_(*t*_*j*_) where *R*_*H*_(*t*_*j*_) is the number of recovered individuals. *S*_*H*_(*t*_*j*_) is given by Eq (6) with

$$C_{H}\left(t_{j}\right)=C_{H}\left(t_{j-1}\right)+E\left[\hat{P}\left(k,t_{j}\right)\right]$$ (10)

where $E[\hat{P}\left(k,t_{j}\right)]$ is the expected number of spillover events during the time-interval [(*j* − 1)*τ*, *jτ*], as $E\left[\hat{P}\left(k,t_{i}\right)\right]={\hat{\lambda }}_{i-1}\tau$, thus we have

$$\begin{array}{ll} C_{H}(t_{j}) & =C_{H}(t_{j-1})+I_{H}^{zoon}+I_{H}^{h-h}\\ I_{H}^{zoon} & =\overset{\text{zoonosis}}{\overset{︷}{\left[N_{H}-C_{H}(t_{j-1})\right]\eta_{R}(N_{R})Pr_{R}(N_{R})\chi_{R}\tau }}+\\ I_{H}^{h-h} & =\overset{\text{human}-\text{to}-\text{human}}{\overset{︷}{\left[N_{H}-C_{H}(t_{j-1})\right]\eta_{H}(N_{H})\frac{I_{H}(t_{j-1})}{S_{H}(t_{j-1})+I_{H}(t_{j-1})+R_{H}(t_{j-1})}\chi_{H}\tau }}\\ & \;\text{until}\;N_{H}\geq C_{H}(t_{j-1}) \end{array}$$ (11)

$C_{H}^{zoon}\left(t_{j}\right)=\sum_{j}I_{H}^{zoon}\left(t_{j}\right)$ represents the cumulative number of infections up to time *t*_*j*_ due to zoonotic spillover and $C_{H}^{h-h}\left(t_{j}\right)=\sum_{j}I_{H}^{h-h}\left(t_{j}\right)$ represents the cumulative number of infections up to time *t*_*j*_ arising from human-to-human transmission. The model requires the further condition:

$$\begin{array}{ll} I_{H}(t_{j}) & =C_{H}(t_{j})-\sum_{i}[R_{H}(t_{j})+D_{H}(t_{j})]\\ R_{H}(t_{j}) & =R_{H}[t_{j-1}]+\gamma_{r}I_{H}[t_{j-1}]\tau \\ D_{H}(t_{j}) & =D_{H}[t_{j-1}]+\gamma_{d}I_{H}[t_{j-1}]\tau \end{array}$$ (12)

where *D*_*H*_(*t*_*j*_) is the disease induced mortality, *γ*_*r*_ and *γ*_*d*_ are the recovery and mortality rates respectively.

The model Eqs (9)–12 can be interpreted as an immigration-birth process [16] where the immigrants, *i.e.* zoonotic spillovers, arrive according to a Poisson process with rate $\hat{\lambda }\left(t_{j}\right)$. Each immigrant produces ‘offspring’, which by analogy is really new infections from human-to-human transmission leading to a stuttering chain, according to a rate which is dependent on past events. The model is a mixture of a self-exciting process (new cases generate subsequent cases (offspring)) and a self-correcting process (due to depletion of susceptibles). We refer to this type of processes as ‘zoonotic spillover with human-to-human transmission’ (in mathematical terms ‘Poisson with Feedback’). We also considered the case when the rate $\hat{\lambda }\left(t_{j}\right)$ is drawn from a gamma-distribution, *i.e.* ‘zoonotic spillover with human-to-human transmission when random effect in the rate are important’ (mathematically ‘Poisson-Gamma Mixture with Feedback’, Table S1 in S2 Text).

The probability $\hat{P}\left(k,t_{j}\right)$ at time *t*_*j*_ in Eq (9) can be iteratively calculated by replacing the susceptible and infected, *S*_*H*_ and *I*_*H*_, with their explicit expressions given in Eqs (6), (10)–12 estimated at the previous time *t*_*j*−1_. The contribution of human-to-human transmission at any time *t*_*j*_, *Q*(*t*_*j*_), can be readily calculated by comparing the cumulative number of infections due to zoonotic transmission terms to those due to human-to-human transmission in Eq (11), for example by studying the quantity:

$$Q\left(t_{j}\right)=\frac{C_{H}^{h-h}\left(t_{j}\right)}{C_{H}\left(t_{j}\right)}$$ (13)

To simplify the notation, we use the symbols *ζ* = *Pr*_*R*_(*N*_*R*_)*χ*_*R*_ *η*_*R*_ and *κ* = *χ*_*H*_ *η*_*H*_ for the overall unknown parameters, and refer to these as ‘zoonotic exposure’ (which incorporates the host infection prevalence) and ‘effective human exposure’ respectively. We also define the forces of infection from animal or human source as Λ_*R*_ = *N*_*Humans*_ *Pr*_*R*_(*N*_*R*_)*χ*_*R*_ *η*_*R*_ and Λ_*H*_ = *N*_*Humans*_ *Pr*_*H*_(*N*_*H*_)*χ*_*H*_ *η*_*H*_ respectively.

Variation in the population size *N*_*H*_ was also considered by discussing how the analytical solutions for the cumulative number of infections scales with the population size and by analysing predictions for *N*_*H*_ = 1000 and *N*_*H*_ = 2000 (S7 and S10 Texts, for the value of the parameters used in the numerics see Table 1 and Table S1 in S6 Text).

**Table 1:** Value of the parameters used in the numerics. See List of Symbols and Glossary in the Supporting Information, S1 Text, for further details.

Parameter	Value	Notes
*N*_*H*_ (Human Population Size)	1000	Unless stated otherwise
*N*_*H*_	2000	Fig S1 in S10 Text
*γ*_*r*_ (Recovery rate)	0.03	Calculated as *γ*_*r*_ = *P*_*recov*_/*T*_*illness*_, where *T*_*illness*_ = 14 days is based on typical period of illness and *P*_*recov*_ = 0.46 is the proportion of patients from KGH who recover. The situation *γ*_*r*_ = 0. was also considered for illustrative purposes.
*γ*_*d*_ (Disease induced mortality rate)	0.04	Calculated as *γ*_*d*_ = *P*_*death*_/*T*_*illness*_, where *P*_*death*_ = 0.54 is the proportion of patients from KGH who died. The situation *γ*_*d*_ = 0. was also considered for illustrative purposes
*κ* (Effective human exposure)	0.02	As imposed in all the ABM unless stated otherwise
*κ*	0.01	As imposed in the ABM in Fig 4
*κ*	median = 0.008338 mean = 0.008364 SD = 0.0006919	Estimated from MCMC, Fig 4
*κ*	median = 0.07395 mean = 0.07383 SD = 0.001549	Estimated from MCMC, Fig 5a
*κ*	median = 0.01868 mean = 0.01863 SD = 0.000947	Estimated from MCMC, Fig 5b
*ζ* (Zoonotic exposure)	0.05	As imposed in all the ABM
*ζ*	median = 3.527*e*−05 mean = 3.651*e*−05 SD = 8.122*e*−06	Estimated from MCMC, Fig 5a
*ζ* slope before 15/03/11	median = 2.245*e*−06 mean = 2.249*e*−06 SD = 1.776*e*−07	Estimated from MCMC, Fig 5b
*ζ* slope after 15/03/11	median = 1.477*e*−06 mean = 1.476*e*−06 SD = 2.626*e*−07	Estimated from MCMC, Fig 5b
*r* (Parameter describing Gamma / Negative-Binomial distributions)	0.035	Eq (S2) in S3 Text, Fig 2
*θ* (Parameter describing Gamma / Negative-Binomial distributions)	1/7	Eq (S2) in S3 Text, Fig 2

### ABM to compare predictions of model with independent simulations

We considered a set of *N*_*H*_ agents. Each agent being in one of four possible categories: susceptible, infected, recovered or dead. At any time step, susceptible agents can transit to the infected category, while infected agents can either recover or die. This is essentially a Bernoulli trial, *e.g.* a random process with exactly two possible outcomes. The transition from the susceptible to the infected status is therefore mimicked by simulating, at any time *t*_*j*_, a number of Bernoulli trials (*S*_*H*_(*t*_*j*_) or *N*_*H*_ if we assume no depletion of susceptibles) with probability given by the appropriate rate divided by the number of trials. For instance, if we considered spillover and human-to-human transmission with depletion of susceptibles, the probability is $\hat{\lambda }\left(t_{j}\right)\tau /S_{H}\left(t_{j}\right)$. This choice ensures that, at any time *t*_*j*_, if the number *S*_*H*_(*t*_*j*_) is large, then the corresponding set of Bernoulli trials are well approximated by a Poisson process with rate $\hat{\lambda }\left(t_{j}\right)\tau$. Similarly, infected agents die or recover by simulating *I*_*H*_(*t*_*j*_) statistically independent Bernoulli trials with probabilities *γ*_*d*_*τ*/*I*_*H*_(*t*_*j*_) and *γ*_*r*_*τ*/*I*_*H*_(*t*_*j*_) respectively.

## Results

The importance of mathematical modelling to elucidate the complexity of infectious disease dynamics and to indicate new approaches to prevention and control is widely accepted (see *e.g.* [17]). The task is not free of challenges, especially for emerging diseases [18]. This is further complicated by abiotic factors such as land use change [4] and social difference demonstrating how risks are not generalisable [19]. Here, we start proposing some measures for the risk of zoonotic spillover and their link with drivers of transmission. Then we present predictions for the model compared with predictions from an independent ABM. Finally we apply the model to LF data, illustrating important challenges and knowledge gaps.

### Suitable measures for the risk of spillover events and their dependence on the drivers of transmission

The mean time between two spillover events and the probability of observing *k* spillovers during a certain time *τ* are suitable measures for the risk of cross-species transmission that naturally arise from the present mathematical framework. Based on the findings above, the risk of a spillover event can be represented by a discrete probability distribution, which can be generally described by a negative binomial distribution. This is fully identified by the mean and variance, empirically inferred or calculated from the mean and variance associated with the rate of infection *λ* as displayed in Table S1 in S3 text.

In some situations, we know how the exposure to the reservoir and its infection prevalence depend on the abundance of the reservoir *N*_*R*_. For example, it is reasonable to expect the exposure *η*_*R*_(*N*_*R*_) is proportional to the reservoir abundance *N*_*R*_. The dependence of the infection prevalence on *N*_*R*_ can also be inferred for many regimes at the endemic equilibrium, *e.g.* frequency and density dependent Susceptible Infected Removed (SIR), Susceptible Exposed Infected Removed (SEIR), etc. models (see Table S1 in S4 Text). For these cases, calculation of the mean and variance *μ*_*λ*_ and $\sigma_{\lambda }^{2}$ is straightforward. In Table S1 in S4 Text, we consider four illustrative scenarios. In many situations the mean risk of spillover increases with the size of the human population *N*_*H*_. The associated variance, however, increases with the square of *N*_*H*_. The dependency on the reservoir abundance *N*_*R*_ is in general more complicated. For instance, in scenario 1 the variance in the risk of spillover, $\sigma_{\lambda }^{2}$, increases with the square of the *N*_*R*_. In contrast, in scenario 2 the variance $\sigma_{\lambda }^{2}$ is not affected by the abundance *N*_*R*_, while in scenario 4 it reaches an asymptotic value for large *N*_*R*_.

### Zoonotic spillovers with constant number of susceptibles

For pure zoonotic spillovers, there is no human-to-human transmission, therefore the rate of infection is not affected by the number of humans already infected. In some cases, variation in the number of susceptibles can be ignored, for example when the impact of immunity and/or mortality is negligible compared to the total population. In this case, every spillover event is independent of previous spillover events. Furthermore, the rate of infection is itself a stochastic quantity as random differences are expected from village to village and from time to time. If these stochastic differences are small, then the rate of infection can be well approximated by its mean value and the distribution of zoonotic spillover described by the well-known Poisson distribution. These stochastic fluctuations, however, can be important; in this case the distribution of zoonotic spillovers is better described by the so-called negative binomial distribution (Eq (S2) in S3 Text, which arises from simple Poisson processes after incorporating stochasticity in the rate of infection given that the distribution of the rates can be well approximated by a gamma-distribution). In this case, the variance of the number of zoonotic spillover events is always larger that their mean value, which is over-dispersion.

**Fig 2:**
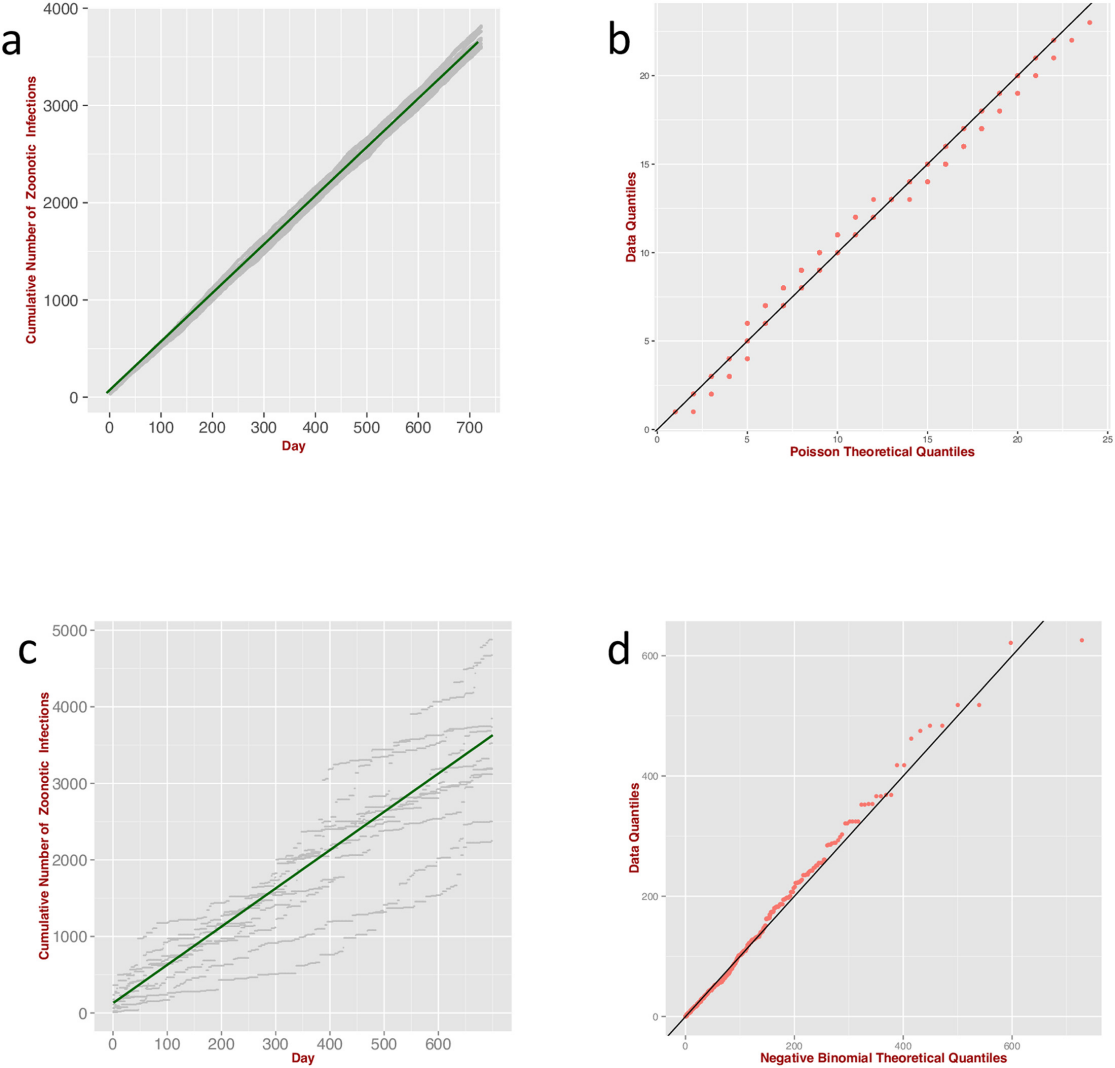
Comparison with ABM I. (**a**) Cumulative number of zoonotic infections generated by the ABM (10 independent runs, grey points) for the case when the rate of infection is not affected by the number of humans already infected (no human-to-human transmission) or by depletion of susceptibles (’Simple Poisson’ model). According to model (1) the mean cumulative number of zoonotic infections grows linearly with *λ* (green line). (**b**) Quantile-Quantile plot of the distribution of infections, generated by the ABM compared with the theoretical Poisson distribution. (**c**) As in panel a, but the rate of infection is also subjected to random variation (‘Poisson-Gamma-Mixture’ model); the green line represents the mean cumulative number of zoonotic infections as in panel a. (**d**) Quantile-Quantile plot of the distribution of infections generated by the ABM compared with the theoretical negative binomial distribution.

Accordingly, we ran the ABM to generate zoonotic infections by simulating *N*_*H*_ random experiments (Bernoulli trials) with transition probability proportional to the force of infection from an animal source (*i.e.* Λ_*R*_*τ*/*N*_*H*_, see Table S1 in S5 Text). Fig 2 shows the cumulative number of zoonotic infections generated by the ABM compared with the corresponding theoretical model (expressed by Eq (1) or Eq (S2) in S3 Text, when random effects in the rate of infection become important). As expected, the profile for the cumulative number of occurrences averaged over the multiple stochastic realisations is linearly increasing with time with the slope given by the mean rate of infection. When the rate of infection is also stochastic, *e.g.* because the outbreaks occurred in different regions with different eco-epidemiological and socio-economic factors, larger deviations from the average profile are observed. This is the typical situation when the available data are aggregated at the national level without distinguishing the specific local factors.

### Zoonotic spillover with depletion of susceptibles

In many situations, the contribution of net immigration, births and deaths (other than infection-induced) to the human population size is negligible, at least for short time-scales. Still, once a spillover occurs, the infected individual might either recover or die, but will never transit back to the susceptible category. Thus we considered the situation when the total number of individuals is fixed, but the number of susceptibles is decreasing due to the accumulation of spillover events resulting in immunity and/or mortality. As the number of infected increase, the pool of susceptibles decreases reducing the rate of new infections; in other words the process is ‘self-correcting’ (Eqs (5)–(8) or their generalization when random effects in the rate of infection become important).

**Fig 3:**
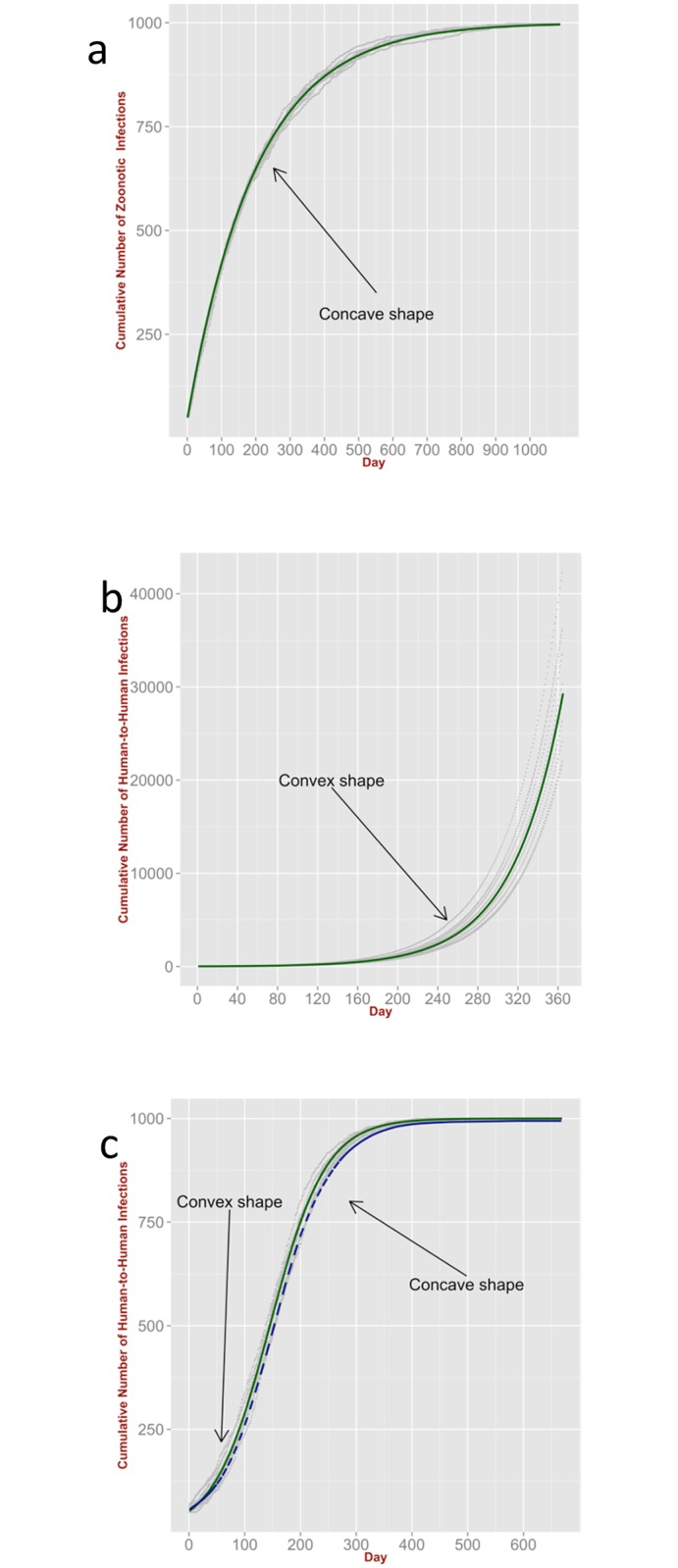
Comparison with ABM II. (**a**) Cumulative number of infections generated by the ABM (10 independent runs, grey points) for zoonotic spillover with depletion of susceptbibles model (‘Self-Correcting Poisson’); its analytical solution (green line), is given by Eq (S3) in S7 Text. (**b**) Cumulative number of infections arising from human-to-human transmission generated by the ABM (10 independent runs, grey points) and no depletion of susceptibles (‘Poisson with Feedback’ model). In the special case of no mortality and no recovery, its analytical solution (green line), is an exponential function (Eq (S10) in S7 Text). (**c**) Cumulative number of infections generated by the ABM (10 independent runs, grey points) for zoonotic spillover with human-to-human transmission and depletion of susceptibles (‘Poisson with Feedback’ model). The dashed blue line represents the mean cumulative number of infections; the special case of no mortality and no recovery is represented by the green line (Eq (S10) in S7 Text).

Accordingly, we ran the ABM to generate zoonotic infections by simulating a number of Bernoulli trials, with number of trials being equal to the time-varying number of susceptibles, and transition probability proportional to the force of infection from animal an source (*i.e.* Λ_*R*_*τ*/*S*_*H*_, see Table S1 in S5 Text. Note the force of infection is time-dependent as the number of susceptibles is changing). Fig 3a shows the cumulative number of zoonotic infections generated by the ABM compared with the theoretical model (Eq (5), see also the analytical solution in S7 Text, for the particular case when the mortality and recovery rates are zero).

As expected, a key effect of incorporating depletion of susceptibles in the model is that the average cumulative number of occurrences always results in a concave (*i.e.* downward) function, provided that there is no birth/immigration of new susceptibles and no temporal variation of the exposure. This is because the rate at which spillover events occur decreases with time and the average size of the jumps in the sample path becomes smaller and smaller. Over time, the profile asymptotically approaches the size of the human population *N*_*H*_ (here set to *N*_*H*_ = 1000 unless stated otherwise). This is more pronounced for high values of the zoonotic force of infection Λ_*R*_.

### Human-to-human transmission and stuttering chain

The ability of a pathogen to transmit between people enables the generation of chains of infection. Fig 3b shows the cumulative number of infections due to only the human-to-human route of transmission. The infections are generated by the ABM by simulating *N*_*H*_ Bernoulli trials with transition probability proportional to the force of infection from human source (*i.e.* Λ_*H*_*τ*/*N*_*H*_ Table S1 in S5 Text).

The predictions are compared with the theoretical model (Eq (9)) with the conditions of no zoonotic spillover and no mortality or recovery. A human infection triggers new infections that, in turn, generate other new infections. In other words the process is ‘self-exciting’ and the cumulative number of infections increases exponentially with rate equal to the effective human exposure (*κ*, Eq (S10) in S7 Text). The presence of zoonotic spillover events leads to a qualitatively similar behaviour, resulting in convex (*i.e.* upward) average profiles for the cumulative number of infections with no upper bound (S8 Text). This because the rate of infection increases as the number of infections increase.

In general, both effects, self-correction due to depletion of susceptibles and self-excitation due to the impact of past infections on new chains of human-to-human transmission, are expected to play a role. The combined effects lead to an average profile for the cumulative number of occurrences that is initially convex until the depletion of susceptibles dominates the dynamics. This can be seen in Fig 3c which shows the cumulative number of infections for the combined ‘zoonotic and human-to-human’ model. The infections were generated by the ABM by simulating Bernoulli trials (with the number of trials equal to the time-varying number of susceptibles), with transition probability proportional to the force of infection from either animal or human source (*i.e.* Λ_*R*_*τ*/*S*_*H*_ or by Λ_*H*_*τ*/*S*_*H*_, Table S1 in S5 Text). The predictions are compared with the corresponding theoretical model (Eq (9)). As expected, the cumulative number of infections increases as an S-shape function asymptotically approaching the human population size *N*_*H*_ (exactly as a logistic function if there is no mortality or recovery, Eq (S10), in S7 Text).

### Estimating the relative contributions of zoonotic spillover and human-to-human transmission

**Fig 4:**
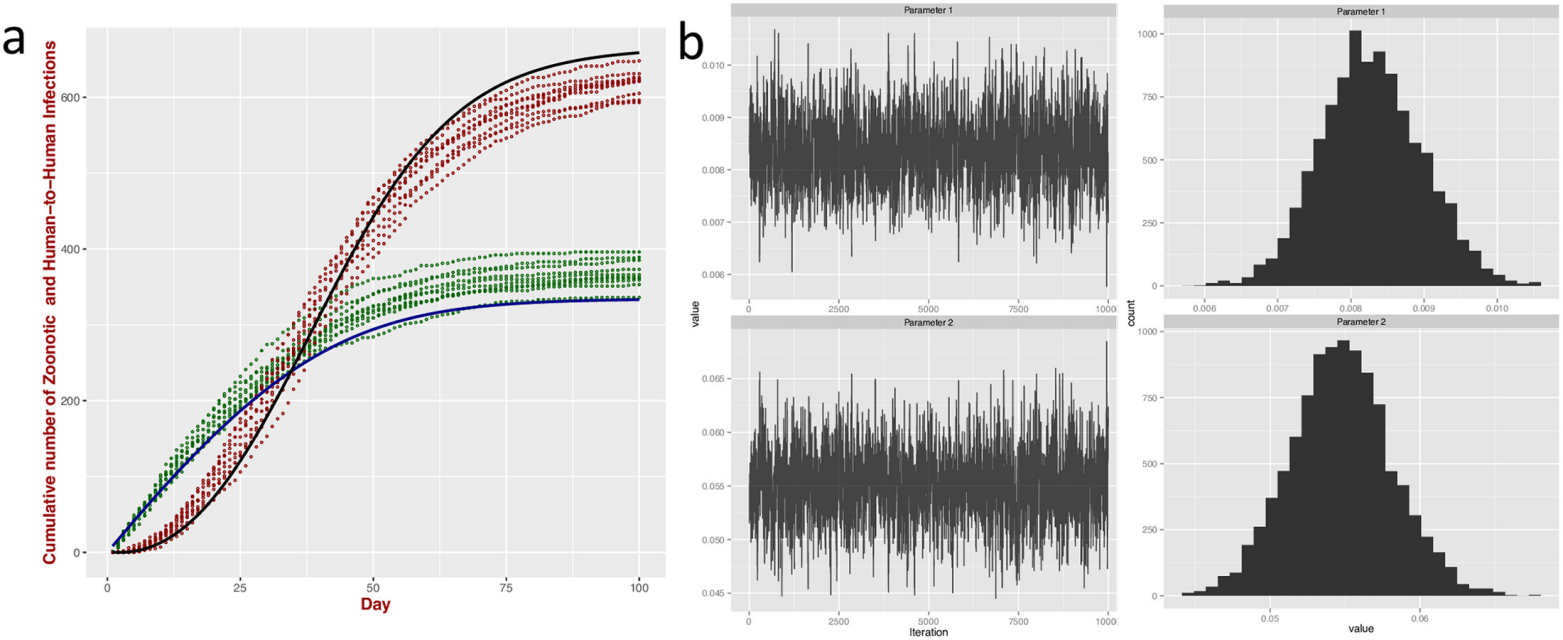
Estimating the relative contributions of zoonotic spillover and human-to-human transmission. Comparison with ABM III.(**a**) Cumulative number of infections for zoonotic spillover with human-to-human transmission and depletion of susceptibles (‘Poisson with Feedback’ model) generated by the ABM (10 independent runs). The green and red points represent cumulative infections arising from zoonotic and human-to-human transmission respectively. The continuous blue and black line represent the analytical solutions and the isolated contributions of zoonotic, $C_{H}^{zoon}$, and human-to-human transmission, $C_{H}^{h-h}$, recalculated with parameters (median values of *ζ* and *κ*, right panel) estimated from the MCMC. (**b**) Traceplot of the time series and histogram of the two parameters: *ζ* (median 0.008338, mean 0.008364, SE 0.0006919, bandwidth 0.0001162) and *κ* (median 0.055118, mean 0.055134, SE 0.0033703, bandwidth 0.0005541) number of iterations 10000, burning time 1000, thinning interval 1. The small discrepancy between the parameters and the ones imposed in the ABM (respectively 0.05 and 0.01) is expected as the ABM simulates Bernoulli trials rather than Poisson processes. Full agreement is expected when the number of trials approaches infinity.

Knowing the zoonotic exposure and effective human exposure, we can estimate the relative contributions of zoonotic spillover and human-to-human transmission, (more precisely, by substituting the values of the two exposures *ζ* and *κ* in Eq (13)). In general these exposures are not known, but can be estimated *via* common statistical techniques, such as Markov Chain Monte Carlo (MCMC). To validate the methodology, we ran the ABM for the combined zoonotic and human-to-human model as described in the above section and counted the number of infections arising from zoonotic transmission and those from human-to-human transmission. All the ABM-simulated infections (with no distinction of the route of transmission) were used as input into MCMC estimation [20, 21] of the zoonotic exposure rate and effective human exposure rate, which are otherwise unknown (*i.e.* the parameters *ζ* and *κ*). The MCMC-inferred parameters were used to calculate the cumulative number of infections due to zoonotic spillover and those due to human-to-human transmission (*i.e.* $C_{H}^{zoon}$ and $C_{H}^{h-h}$, according to Eq (10)) and compared with the corresponding cumulative number of infections generated from the ABM (Fig 4). There is a small discrepancy between the MCMC-inferred parameters and the ones imposed in the ABM (the medians of the two estimated parameters were respectively 0.055 and 0.008 *vs* 0.05 and 0.01). This is expected as the ABM simulates Bernoulli trials rather than Poisson processes and the discrepancy decreases with the number of simulated trials. The prediction improved for larger number of trials (S9 Text). Of course full agreement is expected when the number of trials approaches infinity. For the range of simulations considered here, the relative contributions of zoonotic spillover and human-to-human transmission are not affected by the human population size *N*_*H*_ (Fig S1 in S10 Text).

### Lesson learned from the application to Lassa Fever

**Fig 5:**
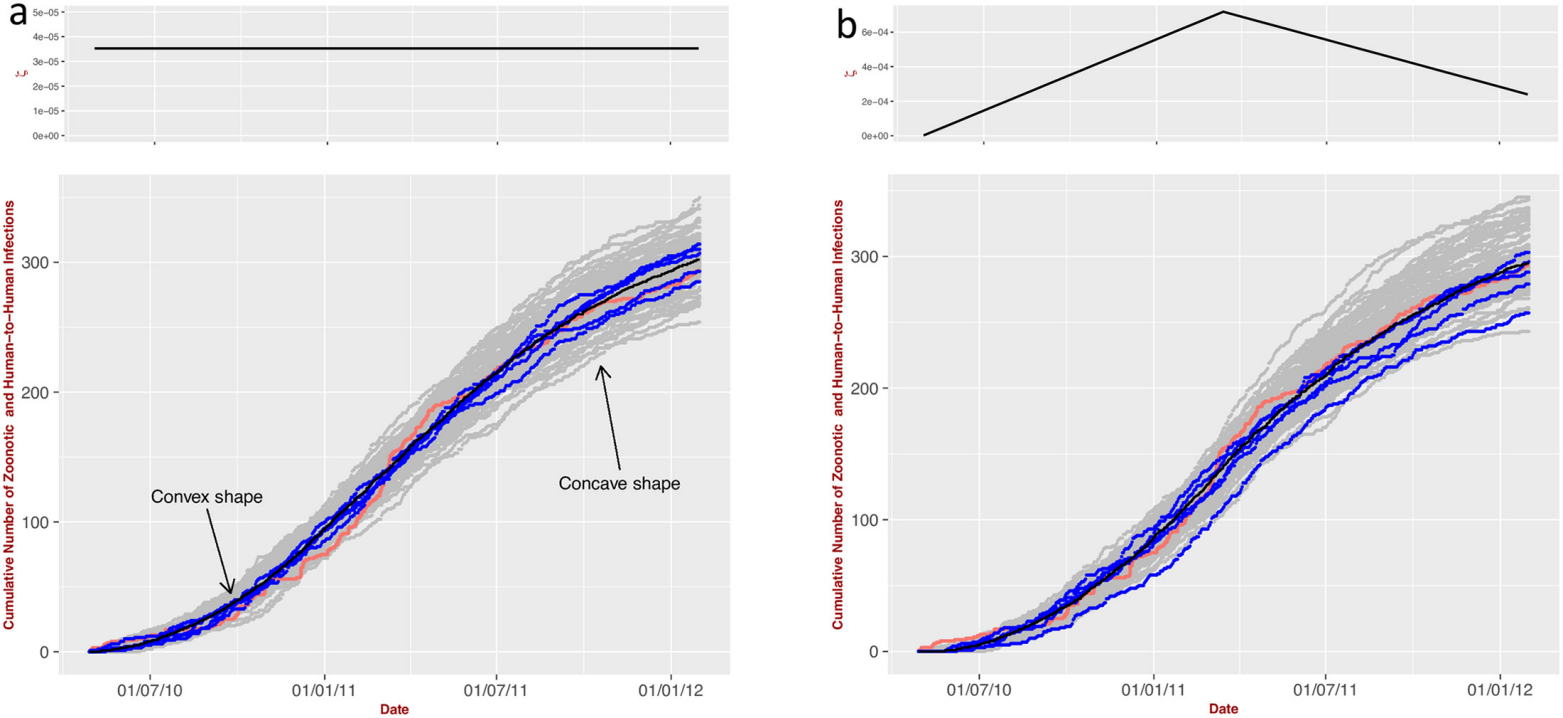
Application to LF. (**a**) Predicted cumulative number of zoonotic and human-to-human infections (governed by ‘Poisson with Feedback’ model, Eq (12)) for constant zoonotic exposure and (**b**) As in (a), but exposure to zoonotic LASV is governed by an piecewise linear trend in exposure to zoonotic LASV (*i.e.* the piecewise zoonotic exposure *ζ* is linearly increasing up to March 2011 followed by a decrease up to January 2012). The parameters are optimized with the data from KGH (red line) by employing MCMC (number of iterations 50000, burning time 1000, thinning interval 1). The grey dots represent 100 independent stochastic realisations; 5 five random examples of which are visualized in blue lines. The black line represents the cumulative number of occurrences averaged over the 100 multiple stochastic realisations.

It is instructive to show some challenges encountered from the application to LF. The key problem is the lack of information on the temporal dependency of the zoonotic exposure (*i.e.* the parameter *ζ*). We therefore considered two simple scenarios. Firstly we assumed a constant zoonotic exposure. Secondly, we allowed its variation in a piecewise linear (triangular) fashion (Fig 5) with the highest peak in March, corresponding to the hottest month in Sierra Leone. This choice is, perhaps, the simplest way to capture variation in the drivers of transmission such as temperature that might affect the abundance and prevalence of the rodents or human mobility. For simplicity we considered only two changes in the slope of this function during the time of the study; this is sufficient for the illustrative purposes of this exercise.

Fig 5 shows the cumulative number of occurrences for the the combined zoonotic and human-to-human model (see Fig S1 in S11 Text, for the corresponding model with a stochastic rate of infection) for constant and piecewise linear variation of the zoonotic exposure. In both situations, the unknown parameters were optimized with the KGH data using an MCMC approach in R [20, 21].

A qualitative inspection shows that both predictions are compatible with the empirical data. The one with constant parameters, however, requires an exceptionally large contribution of human-to-human transmission (≈ 90%) [11]. For the second scenario, agreement with the data requires a positive increase of zoonotic exposure *ζ* followed by a decrease after March 2011 resulting in approximately 22% of cases due to human-to-human transmission. This value, however, is no longer invariant by the human population size; further testing with different values of *N*_*H*_ resulted in different proportions of human-to-human contribution.

A rigorous selection of the two models based on information criteria is problematic. In the current Bayesian context, the Watanabe-Akaike Information Criterion (WAIC) [22, 23] or the Deviance Information Criterion (DIC) [23, 24] appear as ideal tools, at least at first glance. Their suitability, however, is questioned in our specific situation. First, the time series of the number of Lassa cases violates the assumption of independence, which is an essential constraint for WAIC [23]. Lack of independence in the data appears to be a limitation for DIC too [23]. Furthermore, DIC is not appropriate for model selection with mixture models [23], which is the case here as the model comprise multiple populations, *i.e.* the set of individuals infected *via* a zoonotic route and those *via* human-to-human transmission at each time step. In addition, one of the parameters (the zoonotic exposure *ζ*) is time-dependent in one of the models (Fig 5). We are not aware of a rigorous, systematic assessment of the different information criteria in the presence of time-dependent parameters. Finally, an ideal information criterion should select the *true* model, when this is in the model set, and the *closest* one otherwise. This task does not appear to be always achieved by the many information criteria available in the literature. Here, we presented two exemplar models, but in the *true* model, the functional shape of the zoonotic exposure might be different from being constant or piecewise linear, also the effective human exposure might not be constant. For all these reasons, we think that it is more prudent to postpone any conclusion until more accurate data on exposure rates, rodent infection prevalence and reporting bias become available. Nevertheless, for indicative purposes we present the BIC scores for the two models (*BIC* = 30973.96 and *BIC* = 1228.164 respectively). Thus, given the above caveats, the model allowing a temporal variation in zoonotic exposure performs better that the one with constant exposure. This further suggests the importance of solid research in measuring and quantifying exposure.

Temporal variations in rodent abundance and LF virus prevalence were also introduced into the model according to the seasonal patterns observed in West Africa for *M. natalensis* captured inside houses and in the proximity of cultivation [25]. Using these data, the model predictions are not able to capture the initial convex shape in the cumulative number of spillovers (S12 Text).

## Discussion

Based on a systematic review of the literature, Lloyd-Smith *et al.* [3] pointed out that models incorporating spillover and stuttering transmission are rare. The authors found that only 2% of studies of directly-transmitted zoonoses included a mechanistic model of spillover transmission, while stuttering transmission was modelled only in approximately 4% of all studies. Here we developed a unified theoretical framework with the aim of filling this gap. Both zoonotic spillover and stuttering chains can be governed by arrival processes and modelled as generalized Poisson processes, with zoonotic spillover being a particular case of the general model when the probability of human-to-human transmission is null. Although the initial motivation of our work focused on spillovers and stuttering chains (basic reproductive number *R*_0_ < 1), there is no theoretical or practical impediment to use the model beyond the sub-critical regime (*R*_0_ > 1). Indeed the effective reproductive number for Lassa fever data is larger than one [11] (for the effective reproductive number for the simulation generated by the ABM see S13 Text). The theoretical unification of these processes is not a mere question of mathematical elegance, it is critically important for a meaningful comparison of the different stages of disease propagation.

Disentangling the contribution of animal-to-human from human-to-human transmission is of crucial importance to inform appropriate control measures. The shape of the cumulative number of occurrences can provide indications of the modes of transmission. A concave, saturating profile is an expected outcome due to depletion of susceptibles. In contrast, a convex region in the profile of cumulative number of occurrences suggests that human-to-human transmission plays an important role. Alternative explanations are possible. A convex shape in the cumulative number of occurrences might arise from temporal variations in the model parameters (*e.g.* probability of contact between humans and rodents, infection prevalence in rodents, infection-response efficiency) and/or in the human population size.

A fundamental gap in our current knowledge is the mechanisms governing the transition from spillover to stuttering chain to sustained transmission. Stuttering and established human-to-human transmission require the pathogen to have the ability to transmit from human-to-human resulting in a non-zero value for the parameter *χ*_*H*_ (the product of the probability that the virus is excreted from a person and the probability that a person acquires infection when exposed to the virus). Non-biological factors, however, may be involved in the shift from one stage to another. For example, in a sparse population with limited exposure to the reservoir, the disease can rapidly die out due to depletion of susceptibles and/or because the average time between two contacts is longer than the infectious period (stuttering chain scenario). If the size of the human population and/or the frequency of contacts increase, however, then uninterrupted chains of transmission are possible. Thus, the disease switches from a stuttering to a sustained chain of transmission. In the current framework, the conditions leading to this transition can be inferred and quantified by imposing no exposure to the reservoir and studying under which conditions in the parameter values the average solution of Eq (9) results in a fading or in an established non-zero time-series of events.

This work was inspired and guided by the One Health vision: a holistic approach that recognises the inter-connections among human health, animal health and the environment. Accordingly, the model was designed so that a wide range of environmental, biological, ecological, social, economic and political drivers could be readily incorporated. This was done by explicitly expressing the rate of transmission as a function of the constituent factors: the size of the human population, the prevalence of infection in the reservoir host, the probability per time unit of reservoir host-to-human and human-to-human contact, and/or the infection-response efficiency in the human when challenged with the pathogen. For example, complex social, economic and political drivers (*e.g.* demographic pressure, human mobility, etc.) could be translated and quantified in terms of their impact on the typical size of the human population exposed to the disease, *i.e.* the factor *N*_*H*_. Economic and behavioural drivers (*e.g.* the practice of burning fields after harvesting, driving *M. natalensis* towards villages, young boys catching rodents as a ludic activity, seasonal crowding of miners in dwellings) could, once these factors are researched, be expressed in terms of their effects on exposure to disease *i.e.* the factor *η*_*R*_(*N*_*R*_) and *η*_*R*_(*N*_*H*_). Ultimately, complex biological, physical, environmental and social factors can be expressed as factors that can be either measured or quantified via independent models and fed into the current, modular approach or integrated in a Bayesian hierarchical framework.

Being able to infer the likelihood of zoonotic spillover from basic information about the reservoir host and the exposed human population would help to address public health needs and also be of interest to the medical and scientific communities. Our approach addresses this by partitioning the rate of transmission into the product of the constituent factors: the effective human population size at risk, pathogen prevalence in, and human exposure to, the reservoir, and infection-response efficiency. Knowledge of these factors could be gathered, at least in principle, from data collection or other models. For example, despite practical challenges, novel tools for direct or indirect estimation of wildlife abundance/diversity are continuously being developed, *e.g.* remote sensing [26] and public engagement [27]. Also, understanding infection dynamics in reservoir hosts is key to understanding spillover dynamics. Despite logistical and financial challenges, there is an increasing body of research on infection dynamics in wildlife (*e.g.* hantavirus and rodents [28], viral pathogens in African lions [29], viruses in African bats [30], rabies in bats [31]). Quantifying the contact rate of people with the reservoir host and/or other humans is difficult and depends on the mode of transmission, but effective rates of exposure could be estimated from serological data. Of course these factors present a degree of stochasticity, explaining the over-dispersion in many ecological data and indicating that spillover events are governed by Cox processes rather than a simple Poisson process. The probability mass function of spillover events is well described by a negative binomial distribution, suggesting that the rate of transmission is, at least approximately, gamma-distributed (although alternative mechanisms might lead to a negative binomial, S14 Text). Under this assumption, the simple knowledge of mean and variance in the rate of transmission or in the constituent factors are sufficient to completely determine the probability of observing a certain number of spillover events in a particular time-window. Further simplifications are possible when rates of zoonotic exposure and pathogen prevalence in the reservoir host can be explicitly linked to host abundance, as shown for a range of relevant situations discussed in Table S1 in S4 Text. Summary statistics can be readily calculated as the mean rate of spillover events, the mean time between two spillover events and the associated variances. Further theoretical and empirical work in this broad area is essential to enable evidence based reduction of zoonotic disease burden.

### Limitations of this study and opportunities for future research

Application to LF is an interesting example of un-identifiability [32], at least when the analysis was based on a visual inspection alone. Both assumptions (constant and piecewise linear trend in the zoonotic exposure) appear to be equally compatible with the empirical data and the effects of human-to-human transmission can be confounded with those due to temporal variations in the parameters. Nevertheless, the model based on a piecewise linear trend in the zoonotic exposure is the one selected by BIC, although we cannot rule out other models, such as temporally varying zoonotic exposure in a non-linear fashion. Such uncertainty is expected to be removed as soon as more accurate data on actual exposure rates, rodent infection prevalence, spatial distribution of human population size, and further information of reporting bias, become available. For instance, collecting longitudinal human and rodent serological data, at the same location, was the initial objective of the current consortium. However, it was unfortunately hampered by the recent Ebola outbreak. The accuracy of the spatial distribution of human population size is expected to increase and research in health-seeking behaviour is currently conducted in African settings to understand, among other questions, reporting bias. Measuring exposure is in general challenging, although some progress has been made (*e.g.* incidence of arthropod bites in England [33] can be used as proxy for exposure for a range of vector-borne diseases [33]). In general, we believe that inferring exposure requires the combined effort from different types of research, *e.g.* serological surveys, possibly compared with analogue infections from the same reservoir; studies on human behaviour and interaction with the reservoir; mechanistic models to mimic the exposure process. A parallel, interesting avenue of future research would be exploring how the functional form of the zoonotic and effective human exposure affect inference results. It is worth noticing that the proposed framework requires only a general knowledge of the functional form of these quantities (*e.g.* if the temporal profile of the zoonotic exposure is constant, periodic, linear) and not detailed measurements.

In a recent work Andersen *et al.* [34] generated a genomic catalogue of almost 200 LASV sequences from clinical and rodent reservoir samples. Sustained human-to-human transmission would cause a ladder-like genetic structure of the phylogenetic tree which was not observed in their study. Such structure, however, is not expected if most human-to-human transmissions are caused by super-spreaders as recently shown [11]. Understanding super-spreading events is perhaps one of the most compelling scientific challenges in the field of epidemiology. These include molecular techniques to detect them (*e.g.* sequencing the virus in persons known to be infected by a super spreader, quantifying possible differences in the viral load between super-spreaders and non super-spreaders), social science exercises to elucidate behaviour and contact patterns, biological and medical investigation to uncover the physiology of super-spreaders, and mathematical modelling to disentangle the complex interactions of these different factors.

When zoonotic exposure is time-dependent, the predictions are sensitive to the assumed size of the human population. This problem relates to difficult and long-standing questions of spatial scale, and how to enumerate the population at risk in models. In the current framework, the natural spatial scale is the typical spatial range of *M. natalensis*, and the human population size living in the region. Currently we have no detailed information on the location of patients. This is, however, changing as KGH has started to record more accurate information on the address of patients (rather than simply “Kenema” as done in most cases available to us). This information would allow a more realistic meta-population model based on small patches, corresponding to the range of activity of *M. natalensis*, where the human population size is known, the mass action assumption is expected to be more correct, and depletion of susceptibles more relevant. The meta-population model could be improved by allowing immigration/emigration of individuals.

Temporal variations in rodent abundance and LF virus prevalence were introduced in the model according to the seasonal patterns observed in West Africa [25]. The patterns of seasonality in exposure alone, however, cannot explain the particular shape of the cumulative number of cases in KGH data. Furthermore, preliminary social science and rodent ecology data collected by our consortium suggest increased dry season (December to April) exposure linked to intensive cultivation of wetlands for horticulture (see also [4]). The generality of our framework allows the incorporation of a variety of sources of temporal variation.

The impact of stochastic fluctuations on the risk of spillover within the eco-epidemiological systems was not fully studied here. Our work could be further extended to address specific questions like: i) are occasional, large, random bursts in reservoir host infection more likely to spillover into the human population than smaller, but highly correlated, fluctuations? ii) How do model predictions depend on the particular epidemiological model (*e.g.* SI, SEIR, inclusion of reservoir host carrying capacity)? Environmental stochasticity and external periodic drivers (*e.g.* seasonality in the reservoir host population or rainfall) can certainly resonate with the natural frequencies of the eco-system [35] with large effects on transmission dynamics in both reservoir host and the spillover populations. This non-trivial interaction between internal noise and external periodic drivers might explain why evidence of the trophic cascade hypothesis (*e.g.* large amounts of precipitation lead to increased resources, followed by increased rodent abundance and then to increased risk of epizootics and human cases) is so elusive [36].

For simplicity and to demonstrate a proof of concept, our predictions are based on the assumption of uniform mixing and, in most cases, a closed human population; *i.e.* each case from KGH is potentially in contact with each other case and with the rodent population, and no birth or immigration of individuals was allowed. Although this is generally a reasonable assumption when dealing with village communities, as there is high human mobility in Sierra Leone [37, 38], the model ought to be extended to include spatial variability, *e.g.* *via* a meta-population approach and/or linked on spatial environmental and habitat variables [39]. This is an important area where participatory modelling and ethnographic research [37, 40] is much needed to gather information on actual patterns of mobility and social networking, and hence potential contact patterns.

The impact of birth, death and mobility of individual is an an other important topic. Perhaps the simplest scenario is when there is a small immigration of infected into the community (so that the total number of individuals can be approximated as a constant). If this process is governed by a Poisson mechanism, then it is mathematically equivalent to spillover events. This will result in a mere replacement of the zoonotic spillover rate with an ‘effective’ one which incorporate both zoonotic spillover and immigration, with no qualitative change in the findings above. Other scenarios raise more intriguing questions. For example how would the shape of the cumulative number of cases and the proportion of human-to-human transmission change in the presence of a periodic immigration of batches of individual? How is this affected by the size of the batches and the number of infected in each batch? Is there a critical threshold in the flux of infected individuals and the typical time between two arrivals that can lead to persistence of the disease, even in a reservoir-free area? How is the spatial distribution of the disease affected by the particular patterns of human mobility? (*e.g.* by studying traces of bank notes it has been shown that trajectories in human mobility are described by Lèvy walks, see [41] and also [42–44]). These are crucial questions to be addressed in future research.

Measuring the ‘true’ incidence of disease, and therefore morbidity and mortality rates, is a common problem in epidemiology. This includes under-ascertainment arising when not all cases seek healthcare, under-reporting due to failure in the surveillance system, and reporting bias caused in the way the research was conducted [45]. Addressing this important issue is beyond the scope of the current work. We only highlight that in a situation when the zoonotic exposure and the probability of reporting are constant, then reporting bias can have a limited impact on inferring the proportion of human-to-human transmission as we have shown that this is not sensitive to the size of the population *N*_*H*_. Otherwise, marked Poisson processes [46], might be the natural extension of this framework to investigate the effect of reporting bias.

Of course, if KGH, and health authorities in general, could reduce reporting bias it would be highly beneficial to studies such as this. Such improvements include improving methods and increasing funding for contact tracing and rapid diagnostic test development. This is another area where social and ethnographic research is needed to elucidate people’s patterns of reporting and health seeking behaviour for LF, and the social, cultural and economic factors that affect this, so enabling a more accurate assessment of extent and sources of bias.

In conclusion, we developed a conceptually simple, rigorous and transparent framework unifying the fundamental mechanisms of zoonosis with the crucial advantage that, in general, the approach does not require intense numerical computations.

## Supporting Information

S1 Text List of symbols and glossary. (PDF)

S2 Text Overview of different models. (PDF)

S3 Text Relationships between parameters of Poisson and negative binomial distributions. (PDF)

S4 Text Effects of different functional forms of the exposure and prevalence on the rate of spillovers. (PDF)

S5 Text Transition probabilities for the ABM. (PDF)

S6 Text Value of the parameters used in the numerics. (PDF)

S7 Text Analytical solutions for the mean cumulative number of infections for some special cases. (PDF)

S8 Text Cumulative number of infections arising from human-to-human transmission and zoonotic spillover and no depletion of susceptibles generated by the ABM. (PDF)

S9 Text Effect of number of trials. (PDF)

S10 Text Effect of human population size. (PDF)

S11 Text Application to Lassa Fever. Zoonotic Spillover with human-to-human transmission when random effect in the rate are important.
(PDF)

S12 Text Temporal variations in the parameters. (PDF)

S13 Text Effective Reproductive Number for the simulation generated by the ABM (Zoonotic Spillover with human-to-human transmission). (PDF)

S14 Text Alternative mechanisms leading to a Negative-Binomial. (PDF)
