# Supplementary material for: A Unified Framework for the Infection Dynamics of Zoonotic Spillover and Spread
Source: PLoS Negl Trop Dis. 2016 Sep 2;10(9):e0004957. doi: 10.1371/journal.pntd.0004957 (PMC5010258; doi:10.1371/journal.pntd.0004957)
Supplement: S1 Text — (PDF) [file pntd.0004957.s001.pdf]

S1 Text. List of Symbols and Glossary

Diacritical marks (˜) and (ˆ) The signs (˜) and (ˆ) refer, respectively, to the 'Self-Correcting' and 'Poisson with Feedback' models

KGH Acronym for Kenema Governmental Hospital, in Sierra Leone

LASV Lassa fever virus

LF Lassa fever

Stuttering chain/ Stuttering transmission Transmission occurring when human-to-human infections become possible but lead only to self-limiting chains of transmission

Zoonotic spillover/Zoonotic transmission Transmission of pathogens from animals to humans. They are assumed to arise from random and independent contacts with the reservoir.

Infection-response efficiency $\boldsymbol{\chi}_H$ The product of the probability of the virus is excreted from humans and the probability that humans acquire infection when challenged with the virus

Infection-response efficiency $\boldsymbol{\chi}_R$ The product of the probability that the virus is excreted from the reservoir and the probability that humans acquire infection when challenged with the virus

Human population size $\boldsymbol{N}_H$ The total number of people in the suitable system of area $\mathcal{A}$, *e.g.* a village

Rodent abundance $\boldsymbol{N}_R$ The population of rodents in the considered system of area $\mathcal{A}$, *e.g.* a village

Force of infection from animal source $\Lambda_R = N_R Pr_R(N_R)\chi_R\eta_R$ Rate at which susceptible individuals get infected from a animal source.

Force of infection from human source $\Lambda_H = N_H Pr_H(N_H)\chi_H\eta_H$ Rate at which susceptible individuals get infected from a human source.

Zoonotic exposure $\zeta = Pr_R(N_R)\chi_R\eta_R$ Rate at which one susceptible individual get infected after being exposed to the infected reservoir.

Effective human exposure $\kappa = \chi_H\eta_H$ Exposure to other humans rescaled by the infection-response efficiency η_H.

Rate $\boldsymbol{\lambda}$, $\tilde{\boldsymbol{\lambda}}_{\boldsymbol{j}}$, $\hat{\boldsymbol{\lambda}}_{\boldsymbol{j}}$ Expected number of zoonotic spillovers per time unit for the different models. Index j implies that the rate is time-dependent.

$\boldsymbol{C_H(t_j)}$ cumulative number of people who had been infected at any past time during the interval $[0, t_j]$, irrespective of if they recovered or died

$\boldsymbol{C_H^{zoon}(t_j)}$ cumulative number of people who had been infected from animals at any past time during the interval $[0, t_j]$, irrespective of if they recovered or died

$\boldsymbol{C_H^{h-h}(t_j)}$ cumulative number of people who had been infected from humans at any past time during the interval $[0, t_j]$, irrespective of if they recovered or died

$\boldsymbol{D_H(t_j)}$ number of dead in the human population at time t_j.

$\boldsymbol{\eta_R(N_R)}$ A measure of exposure, given by the product $\eta_R(N_R) = \xi(N_R)\mathcal{A}$ where $\mathcal{A}$ is the area of the considered system, *e.g.* a village, and $\xi(N_R)$ is the probability of a contact (direct or mediated) between *a single* member of the human population and the population of N_R rodents, per time unit and area unit

$\boldsymbol{\eta_H(N_H)}$ the probability that *a single* person is in contact with any other member of the human population, per time unit

$\boldsymbol{\gamma_r}$ recovery rate

$\boldsymbol{\gamma_d}$ disease induced mortality rate

$\boldsymbol{I_H(t_j)}$ number of infected in the human population at time t_j.

$\boldsymbol{\mu}$ mean associated with the Negative-Binomial distribution (Eq (S3))

$\boldsymbol{\mu_\lambda}$ mean of the associated Gamma-distribution for the rate λ (Eq (S2) in S3 Text, table S1 in S3 text)

$\boldsymbol{\mu_X}$ mean of the random variable X

$\boldsymbol{N_H}$ human population size.

$\boldsymbol{P(k)}$, $\boldsymbol{\tilde{P}(k, t_j)}$, $\boldsymbol{\hat{P}(k, t_j)}$ Probability of observing k infections (or admissions to KGH) at any time t_j during the interval $[(j-1)\tau, j\tau]$ for the different models. For the 'Simple Poisson' model this probability does not depend on the time t_j

$\boldsymbol{P_{N_H}(N_H)}$, $\boldsymbol{P_{N_R}(N_R)}$, $\boldsymbol{P_{\chi_R}(\chi_R)}$ $P_{N_H}(N_H)$ and $P_{N_R}(N_R)$ are the probability density functions of observing N_R rodents and N_H human in the system. $P_{\chi_R}(\chi_R)$ is the probability density function of the infection-response efficiency χ_R

$\boldsymbol{P_\lambda(\lambda; r, \theta)}$, $\boldsymbol{r}$, $\boldsymbol{\theta}$, $\boldsymbol{\Gamma(r)}$, $\boldsymbol{p}$ $P_\lambda(\lambda; r, \theta)$ is the distribution of the (stochastic) rate λ in Cox model. r, θ and p are the parameters describing a Gamma and a Negative-Binomial distributions. $\Gamma(r)$ is the Gamma function (see Eq (S3))

$\boldsymbol{Pr_R(N_R)}$ infection prevalence in rodents

$\boldsymbol{Pr_H(N_H)}$ infection prevalence in humans

$\boldsymbol{Q}$ proportion of LF cases who contracted the disease from a human source (Eq (13))

$\boldsymbol{R_H(t_j)}$ number of recovered in the human population at time t_j.

$\boldsymbol{S_H(t_j)}$ number of susceptibles in the human population at time t_j.

$\boldsymbol{\sigma_\lambda^2}$ variance associated with the Negative-Binomial distribution (Eq (S3))

$\boldsymbol{\sigma^2}$ variance associated with the Gamma-distribution for the rate λ (Eq (S2) in S3 Text, table S1 in S3 text)

$\boldsymbol{\sigma_X^2}$ variance of the random variable X

$\boldsymbol{t_j}$ discrete time defined by the interval $[(j-1)\tau, j\tau]$ (with $t_j \in [(j-1)\tau, j\tau]$).

$\boldsymbol{\tau}$ time unit
