# Supplementary material for: A Unified Framework for the Infection Dynamics of Zoonotic Spillover and Spread
Source: PLoS Negl Trop Dis. 2016 Sep 2;10(9):e0004957. doi: 10.1371/journal.pntd.0004957 (PMC5010258; doi:10.1371/journal.pntd.0004957)
Supplement: S2 Text — (PDF) [file pntd.0004957.s002.pdf]

S2 Text. Overview of different models. Table S1 present an overview of different models for the probability P that k events occur during a time τ. For all models the probability is described by a Poisson-like distribution $P(k) = \frac{\exp^{-\lambda\tau}(\lambda\tau)^k}{k!}$ with the main difference driven by the form of the rate λ.

Table S1. Overview of different models

Model	Key Mathematical Features	Biological System
**Pure Zoonotic Spillover** Simple Poisson model	λ is a fixed parameter. Equality between mean and variance in the expected number of infections. The cumulative number of infections increases linearly with time	Infection events due to zoonotic spillover only. No depletion of susceptibles and no over-dispersion.
**Time-Dependent Zoonotic Spillover** Simple, Inhomogeneous Poisson model	As in the Simple Poisson model but λ is a function of time.	As in the Simple Poisson model, but it can be extended to situations when the rate is time-dependent, *e.g.* when the rate is deterministically driven by seasonality in the constituent factors.
**Zoonotic Spillover when random effects in the rate are important** Poisson-Gamma Mixture	The rate λ is itself a stochastic variable. If λ is drawn from a gamma distribution the resulting probability P is described by a Negative Binomial.	Infection events due to zoonotic spillover only. No depletion of susceptibles. Over-dispersion in the data.
**Zoonotic Spillover with depletion of susceptibles** Self-Correcting Poisson	The rate λ is explicitly dependent on, and decreasing with, the number of infections that occurred in the past. The temporal profile of the average cumulative number of infections is always concave. It increases non-linearly with time and asymptotically approaches the total number of susceptibles.	Infection events due to zoonotic spillover only. Their number is upper-bounded because of depletion of available susceptibles.
**Zoonotic Spillover with depletion of susceptibles when random effects in the rate are important** 'Self-Correcting Poisson-Gamma Mixture'	As in the 'Self-Correcting Poisson' model but the rate λ is itself a stochastic variable.	Infection events due to zoonotic spillover only. Their number is upper bounded because of depletion of available susceptibles. Over-dispersion in the data.
**Zoonotic Spillover with human-to-human transmission** 'Poisson with Feedback'	λ is explicitly depends on the number of infections that occurred in the past. If there is no depletion of susceptibles, the temporal profile of the average cumulative number of infection is always convex and it indefinitely increases with time. Otherwise it is described by an S-shape curve which asymptotically approaches the total number of susceptibles.	Infection events due to zoonotic spillover and human-to-human transmission.
**Zoonotic Spillover with human-to-human transmission when random effect in the rate are important** 'Poisson-Gamma Mixture with Feedback'	As in the Poisson with Feedback' model but the rate λ is itself a stochastic variable.	Infection events due to zoonotic spillover and human-to-human transmission. Over-dispersion in the data.
