# Supplementary material for: A Unified Framework for the Infection Dynamics of Zoonotic Spillover and Spread
Source: PLoS Negl Trop Dis. 2016 Sep 2;10(9):e0004957. doi: 10.1371/journal.pntd.0004957 (PMC5010258; doi:10.1371/journal.pntd.0004957)
Supplement: S3 Text — (PDF) [file pntd.0004957.s003.pdf]

S3 Text. Relationships between parameters of Poisson and negative binomial distributions. Assuming that the prevalence and exposure can be deterministically inferred from the human and rodent population, Eq (1) ought to be replaced with

$$\begin{aligned} P(k) &= \int \frac{\exp^{-\lambda\tau}[\lambda\tau]^k}{k!} \left[P_{N_H}(N_H) P_{N_R}(N_R)\right. \\ &\quad \left. P_{\chi_R}(\chi_R)\right] \mathrm{d}N_H \mathrm{d}N_R \mathrm{d}\chi_R \\ \lambda &= N_H \eta_R(N_R) Pr_R(N_R) \chi_R \end{aligned} \quad \text{(S1)}$$

where $P_{N_H}(N_H)$ and $P_{N_R}(N_R)$ are the probability density functions of observing N_R rodents and N_H humans in the system. Similarly $P_{\chi_R}(\chi_R)$ is the probability density function of the infection-response efficiency, χ_R.

Of course, the confirmed cases of LF in KGH correspond to a subset of all cases of LF occurring in the region, *i.e.* only the detected cases. Although under-reporting is a major problem in public health, we focus only on the reported cases of LF for which we have data. This is sufficient for the illustrative purposes of the current analysis.

An important example of a Cox process is the case when the rate λ is a gamma-distributed variable, *i.e.*

$$\begin{aligned} P(k) &= \int \frac{\exp^{-\lambda\tau}(\lambda(N_H,N_R,\chi_R)\tau)^k}{k!} P_\lambda(\lambda; r, \theta) \mathrm{d}\lambda \\ P_\lambda(\lambda; r, \theta) &= \frac{\lambda^{r-1} e^{-\frac{\lambda}{\theta}}}{\theta^r \Gamma(r)} \quad \text{for } \lambda > 0 \text{ and } r, \theta > 0. \end{aligned} \quad \text{(S2)}$$

In this situation the Cox process (S2) is described by a negative binomial distribution [1, 2].

$$P(k) = \binom{k+r-1}{k} p^k (1-p)^r \quad \text{for } k = 0, 1, 2, \ldots \quad \text{(S3)}$$

with further conditions that the shape in the gamma-distributed random variable λ is equal to r and the scale $\theta = p/(1-p)$. After some algebra based on well-known properties of the negative binomial distribution, we can present further relationships between some parameters of the negative binomial distribution (including mean μ and variance σ^2 that uniquely determine the distribution) and the mean μ_λ and variance σ_λ^2 of the associated gamma-distribution for the rate λ (Table S1).

Table S1. Relationships between the mean and variance of the negative binomial distribution (μ and σ^2) and the mean and variance of the associated gamma-distribution for the rate λ (μ_λ and σ_λ^2). Here and throughout the symbol μ_X and σ_X^2 represent the mean and variance of the random variable X. The symbol $\lfloor x \rfloor = floor(x)$ is the largest integer not greater than x. Further qualitative properties of the curve describing the risk, *e.g.* mode, skewness and kurtosis, can be extracted directly from the knowledge of the mean and variance. For example, if $\mu_\lambda \leq \sigma_\lambda^2$ then the mode is zero, otherwise the mode increases with the mean μ_λ (almost linearly for large values of μ_λ or small variance σ_λ^2).

**Parameter**	**Relationship with μ and σ^2**	**Relationship with μ_λ and σ_λ^2**
Mean μ		$\mu = \mu_\lambda$
Variance σ^2		$\sigma^2 = \sigma_\lambda^2 + \mu_\lambda$
Parameter p in NB	$p = 1 - \frac{\mu}{\sigma^2}$	$p = \frac{\sigma_\lambda^2}{\sigma_\lambda^2 + \mu_\lambda}$
Parameter r in NB; Shape in gamma-function	$r = \frac{\mu^2}{\sigma^2 - \mu}$	$r = \frac{\mu_\lambda^2}{\sigma_\lambda^2}$
Mode	$\begin{cases} \lfloor \mu + 1 - \frac{\sigma^2}{\mu} \rfloor & \text{if } \mu + 1 - \frac{\sigma^2}{\mu} > 0 \\ 0 & \text{if } \mu + 1 - \frac{\sigma^2}{\mu} \leq 0 \end{cases}$	$\begin{cases} \lfloor \frac{\mu_\lambda^2 - \sigma_\lambda^2}{\mu_\lambda} \rfloor & \text{if } \mu_\lambda^2 > \sigma_\lambda^2 \\ 0 & \text{if } \mu_\lambda^2 \leq \sigma_\lambda^2 \end{cases}$
Skewness	$\frac{2\sigma}{\mu} - \frac{1}{\sigma}$	$\frac{2\sqrt{\sigma_\lambda^2 + \mu_\lambda}}{\mu_\lambda} - \frac{1}{\sqrt{\sigma_\lambda^2 + \mu_\lambda}}$
Excess Kurtosis	$\frac{1}{\sigma^2} + \frac{6(\sigma^2 - \mu)}{\mu^2}$	$\frac{1}{\sigma_\lambda^2 + \mu_\lambda} + \frac{6\sigma_\lambda^2}{\mu_\lambda^2}$

References

1. Greenwood M, Yule GU. An Inquiry into the Nature of Frequency Distributions Representative of Multiple Happenings with Particular Reference to the Occurrence of Multiple Attacks of Disease or of Repeated Accidents. Journal of the Royal Statistical Society. 1920;83(2):255. doi:10.2307/2341080.

2. Daley DJ, Vere-Jones D. Introduction to the Theory of Point Processes. New York ; London: Springer; 1988.
