# Supplementary material for: A Unified Framework for the Infection Dynamics of Zoonotic Spillover and Spread
Source: PLoS Negl Trop Dis. 2016 Sep 2;10(9):e0004957. doi: 10.1371/journal.pntd.0004957 (PMC5010258; doi:10.1371/journal.pntd.0004957)
Supplement: S4 Text — (PDF) [file pntd.0004957.s004.pdf]

S4 Text. Effects of different functional forms of the exposure and prevalence on the rate of spillovers.

Table S1. Effects of different functional forms of the exposure $\eta(N_R)$ and prevalence $Pr_R(N_R)$ on the mean and variance μ_λ and σ^2_λ of the rate of spillovers. C and D are constants.

	$\eta(N_R)$, $Pr_R(N_R)$, λ	μ_λ, σ_λ	**Notes**
Scenario 1	$\eta(N_R) \propto N_R$<br>$Pr(N_R)$ not dependent on N_R<br>$\lambda = CN_H N_R \chi_R$	$\mu_\lambda = C\mu_{N_H}\mu_{N_R}\mu_{\chi_R}$<br>$\sigma^2_\lambda \approx (CN_R\chi_R)^2 \sigma^2_{N_H} + (CN_H\chi_R)^2 \sigma^2_{N_R} + (CN_H N_R)^2 \sigma^2_{\chi_R}$	Exposure proportional to host abundance. Infection prevalence does not depend on host abundance. *E.g.* a scenario described by a frequency dependent SIR model with fixed population at endemic equilibrium [1].
Scenario 2	$\eta(N_R) = \eta_\infty$<br>$Pr(N_R)$ not dependent on N_R<br>$\lambda = CN_H \eta_\infty \chi_R$	$\mu_\lambda = C\mu_{N_H}\eta_\infty\mu_{\chi_R}$<br>$\sigma^2_\lambda \approx (C\eta_\infty\chi_R)^2 \sigma^2_{N_H} + (CN_H\eta_\infty)^2 \sigma^2_{\chi_R}$	Exposure does not depend on host abundance. Infection prevalence does not depend on host abundance. *E.g.* a scenario where the probability of contacts saturates for a large $N_{R\infty}$ asymptotically approaching the value η_∞.
Scenario 3	$\eta(N_R) \propto N_R$<br>$Pr(N_R) = 1 - \frac{D}{N_R}$<br>$\lambda = CN_H(N_R - D)\chi_R$	$\mu_\lambda = C\mu_{N_H}\mu_{\chi_R}[1 - D\mu_{N_R}]$<br>$\sigma^2_\lambda \approx [C(N_R - D)\chi_R]^2 \sigma^2_{N_H} + [CN_H\chi_R]^2 \sigma^2_{N_R} + [CN_H(N_R - D)]^2 \sigma^2_{\chi_R}$	Exposure proportional to host abundance as in scenario 1. Infection prevalence also depends on host abundance. This form of prevalence arises, *e.g.*, from a density dependent SI model with fixed birth rate at endemic equilibrium.
Scenario 4	$\eta(N_R) = \eta_\infty$<br>$Pr(N_R) = 1 - \frac{D}{N_R}$<br>$\lambda = \eta_\infty N_H \left(1 - \frac{D}{N_R}\right)\chi_R$	$\mu_\lambda = \eta_\infty\mu_{N_H}\mu_{\chi_R}\left[1 - D\mu_{[1/N_R]}\right]$<br>$\sigma^2_\lambda \approx \left[\eta_\infty\left(1 - \frac{D}{N_R}\right)\chi_R\right]^2 \sigma^2_{N_H} + \left[\eta_\infty N_H \frac{D}{N_R^2}\chi_R\right]^2 \sigma^2_{N_R} + \left[\eta_\infty N_H\left(1 - \frac{D}{N_R}\right)\right]^2 \sigma^2_{\chi_R}$	Exposure does not depend on host abundance as in scenario 2. Infection prevalence depends on host abundance as in scenario 3.

References

1. Keeling MJ, Rohani P. Modeling Infectious Diseases in Humans and Animals. Princeton University Press; 2008.
