# Supplementary material for: A Unified Framework for the Infection Dynamics of Zoonotic Spillover and Spread
Source: PLoS Negl Trop Dis. 2016 Sep 2;10(9):e0004957. doi: 10.1371/journal.pntd.0004957 (PMC5010258; doi:10.1371/journal.pntd.0004957)
Supplement: S5 Text — (PDF) [file pntd.0004957.s005.pdf]

S5 Text. Transition probabilities for the ABM.

Table S1. Transition Probabilities used in the ABM

Model	Transition Probability	Notes
**Pure Zoonotic Spillover** (Simple Poisson model)	$\Lambda_R\tau/N_H$	the rate of change in the limiting Poisson process is therefore the constant $\lambda(t_j) = \Lambda_R\tau$
**Zoonotic Spillover with depletion of susceptibles** (Self-Correcting Poisson)	$\Lambda_R\tau/S_H$	the rate of change in the limiting Poisson process is therefore the constant $\tilde{\lambda}(t_j) = \Lambda_R\tau$
**Human-to-human transmission** (Self-Exciting Poisson)	$\Lambda_H\tau/S_H$	the rate of change in the limiting Poisson process is therefore the time-dependent $\hat{\lambda}(t_j) = \Lambda_H\tau$
**Zoonotic Spillover with human-to-human transmission** (Poisson with Feedback)	$\Lambda_R\tau/S_H$ and $\Lambda_H\tau/S_H$	the rate of change in the limiting Poisson process is therefore the time-dependent $\hat{\lambda}(t_j) = (\Lambda_R + \Lambda_H)\tau$
