# Supplementary material for: A Unified Framework for the Infection Dynamics of Zoonotic Spillover and Spread
Source: PLoS Negl Trop Dis. 2016 Sep 2;10(9):e0004957. doi: 10.1371/journal.pntd.0004957 (PMC5010258; doi:10.1371/journal.pntd.0004957)
Supplement: S6 Text — (PDF) [file pntd.0004957.s006.pdf]

S6 Text. Value of the parameters used in the numerics.

Table S1. Additional value of the parameters used in the numerics

**Parameter**	**Value**	**Notes**
κ	median 0.0751363, mean 0.07511, SD $2.066e-03$;	Estimated from MCMC, Fig S1 in S11 text,for zoonotic Spillover with human-to-human transmission when random effect in the rate are important (‘Poisson-Gamma Mixture with Feedback’ model)
ζ	median = 0.0000334, mean = 0.00003433, SD = $7.625e-06$	Estimated from MCMC,Fig S4 for ‘Poisson-Gamma Mixture with Feedback’ model
p	median = 0.4743519, mean = 0.4760 , SD = $3.519e-02$	Estimated from MCMC, Fig S1 in S11 text, for ‘Poisson-Gamma Mixture with Feedback’ model
λ	mean = 1.36744186 SD = 0.07975083;	Estimated from maximum-likelihood, Fig S4 for ‘Simple Poisson’ model
r	1.25	Eq (S2) in S3 text,, Estimated from maximum-likelihood, Fig S1 in S11 text, for ‘Poisson-Gamma Mixture’ model
θ	1.091	Eq (S2) in S3 text,, Estimated from maximum-likelihood, Fig S1 in S11 text, for ‘Poisson-Gamma Mixture’ model
