# Supplementary material for: A Unified Framework for the Infection Dynamics of Zoonotic Spillover and Spread
Source: PLoS Negl Trop Dis. 2016 Sep 2;10(9):e0004957. doi: 10.1371/journal.pntd.0004957 (PMC5010258; doi:10.1371/journal.pntd.0004957)
Supplement: S7 Text — (PDF) [file pntd.0004957.s007.pdf]

S7 Text. Analytical solutions for the mean cumulative number of infections for some special cases. Here we present the analytical solutions for the mean cumulative number of infections for the 'Self-Correcting Poisson' model, and for the 'Poisson with Feedback' model with the further conditions $\gamma_d = \gamma_r = 0$

Self-Correcting Poisson model

Equations (5)-(8) can be rewritten as:

$$\frac{C_H(t_j) - C_H(t_{j-1})}{\tau} = [N_H - C_H(t_{j-1})]\,\eta_R(N_R)Pr_R(N_R)\chi_R$$

its continuous counterpart is the differential equation (with $\tau = t_j - t_{j-1} \rightarrow \mathrm{d}t$):

$$\frac{\mathrm{d}C_H(t)}{\mathrm{d}t} = [N_H - C_H(t)]\,\eta_R(N_R)Pr_R(N_R)\chi_R \tag{S1}$$

whose solution is:

$$C(t) = [C(t_0) - N_H]\exp\left[-(t - t_0)\eta_R(N_R)Pr_R(N_R)\chi_R\right] + N_H \tag{S2}$$

Poisson with Feedback model and no depletion of susceptibles

In this case the analogue of equation Eq (11) is:

$$C_H(t_j) = C_H(t_{j-1}) + N_H\eta_R(N_R)Pr_R(N_R)\chi_R\tau + N_H\eta_H(N_H)\frac{I_H(t_{j-1})}{N_H}\chi_H\tau \tag{S3}$$

where the term $[N_H - C_H(t_{j-1})]$is replaced with N_H (as there is no depletion of susceptibles, *i.e.* $N_H >> C_H$) and the infection prevalence in humans is given by $\frac{I_H(t_{j-1})}{N_H}$. If $\gamma_d = \gamma_r = 0$, then (see Eq (12)) $I_H(t_j) = C_H(t_j)$ and Eq (S3) can be rewritten as:

$$\frac{C_H(t_j) - C_H(t_{j-1})}{\tau} = N_H\eta_R(N_R)Pr_R(N_R)\chi_R + \eta_H(N_H)C_H(t_{j-1})\chi_H \tag{S4}$$

(S5)

its continuous counterpart is the differential equation:

$$\frac{\mathrm{d}C_H(t)}{\mathrm{d}t} = N_H\eta_R(N_R)Pr_R(N_R)\chi_R + \eta_H(N_H)C_H(t)\chi_H \tag{S6}$$

whose solution is

$$C_H(t) = C_H(t_0)\exp\left[\eta_H(N_H)\chi_H\,(t - t_0)\right] - \frac{N_H\eta_R(N_R)Pr_R(N_R)\chi_R}{\eta_H(N_H)\chi_H} \tag{S7}$$

Poisson with Feedback model

When $\gamma_d = \gamma_r = 0$, from Eq (12) $I_H(t_j) = C_H(t_j)$ and therefore equation (11) can be rewritten as:

$$\frac{C_H(t_j) - C_H(t_{j-1})}{\tau} =$$
$$[N_H - C_H(t_{j-1})]\,\eta_R(N_R)Pr_R(N_R)\chi_R + [N_H - C_H(t_{j-1})]\,\eta_H\frac{C_H(t_{j-1})}{N_H}\chi_H \tag{S8}$$

its continuous counterpart is the differential equation:

$$
\begin{aligned}
&\frac{1}{a + bC_H(t) - cC_H(t)^2} \frac{\mathrm{d}C_H(t)}{\mathrm{d}t} = \mathrm{d}t \\
&a = N_H \eta_R(N_R) Pr_R(N_R) \chi_R \\
&b = -\eta_R(N_R) Pr_R(N_R) \chi_R + \eta_H \chi_H \\
&c = \frac{\eta_H \chi_H}{N_H}
\end{aligned} \tag{S9}
$$

whose solution is:

$$
\begin{aligned}
&C_H(t) = \frac{BC_1 \exp \delta t - A}{2\left(1 - C_1 \exp \delta t\right)} \\
&\delta = \sqrt{(4ac + b^2)} \\
&A = 4a\sqrt{\left(\frac{1}{4ac + b^2}\right)} + \frac{b^2}{c}\sqrt{\left(\frac{1}{4ac + b^2}\right)} - \frac{b}{c} \\
&B = -4a\sqrt{\left(\frac{1}{4ac + b^2}\right)} - \frac{b^2}{c}\sqrt{\left(\frac{1}{4ac + b^2}\right)} - \frac{b}{c} \\
&C_1 = \frac{2C_H(t_0) + A}{B + 2C_H(t_0)}
\end{aligned} \tag{S10}
$$

Note, if $\eta_R(N_R) Pr_R(N_R) \chi_R = 0$ (no zoonotic spillovers), Eq (S9) reduces to the common logistic equation for population growth

$$
\frac{\mathrm{d}C_H(t)}{\mathrm{d}t} = r\left(1 - \frac{C_H(t)}{K}\right) \tag{S11}
$$

with growth rate $r = \eta_H \chi_H$ and carrying capacity $K = N_H$ whose solution is:

$$
C_H(t) = \frac{N_H C_H(t_0) \exp\left[\eta_H \chi_H \left(t - t_0\right)\right]}{N_H + C_H(t_0)\left(\exp\left[\eta_H \chi_H \left(t - t_0\right)\right] - 1\right)} \tag{S12}
$$
