# Supplementary material for: A Unified Framework for the Infection Dynamics of Zoonotic Spillover and Spread
Source: PLoS Negl Trop Dis. 2016 Sep 2;10(9):e0004957. doi: 10.1371/journal.pntd.0004957 (PMC5010258; doi:10.1371/journal.pntd.0004957)
Supplement: S8 Text — (PDF) [file pntd.0004957.s008.pdf]

S8 Text. Cumulative number of infections arising from human-to-human transmission and zoonotic spillover and no depletion of susceptibles generated by the ABM.

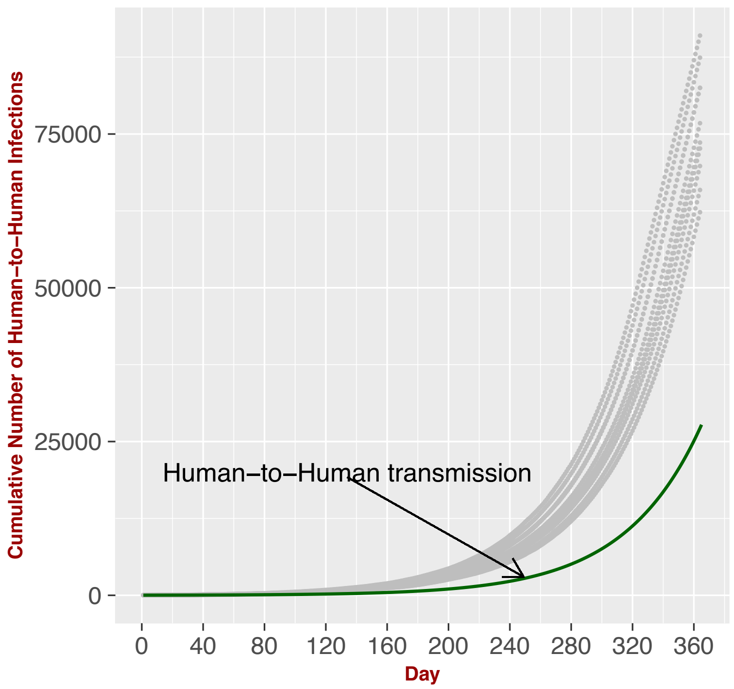

Fig S1. Cumulative number of infections arising from human-to-human transmission and zoonotic spillover ($\zeta = 0.01$ generated by the ABM (10 independent runs, grey points) and no depletion of susceptibles. The green line shows the the special case of human-to-human transmission only.
