# Supplementary material for: A Unified Framework for the Infection Dynamics of Zoonotic Spillover and Spread
Source: PLoS Negl Trop Dis. 2016 Sep 2;10(9):e0004957. doi: 10.1371/journal.pntd.0004957 (PMC5010258; doi:10.1371/journal.pntd.0004957)
Supplement: S9 Text — (PDF) [file pntd.0004957.s009.pdf]

S9 Text. Effect of number of trials.

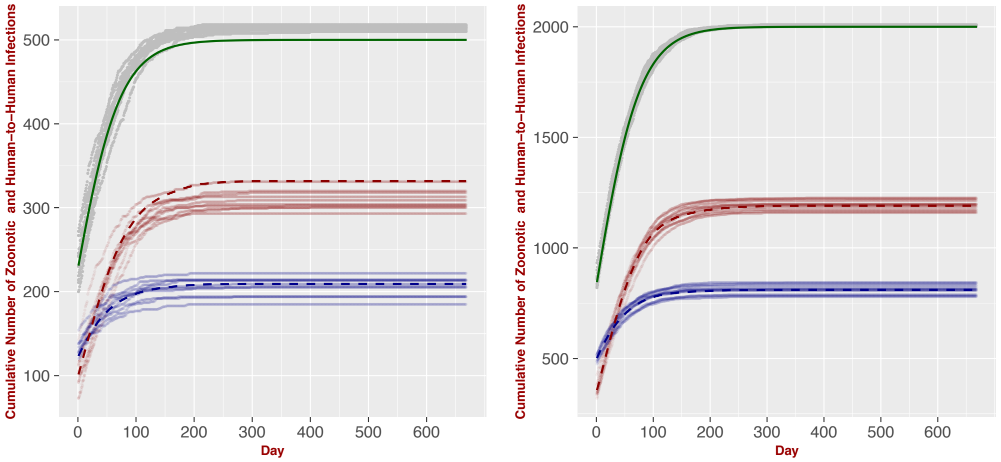

Fig S1. Cumulative number of infections for the 'Poisson with Fedback' model generated by the ABM (10 independent runs). The red and blue points represent cumulative infections arising from zoonotic and human-to-human transmission respectively; the grey points are all cumulative infections altogether. The green line is the analytical solution for the mean cumulative number of infections according to Eq (S10) in the Supporting Information, S7 Text. (**a**) Human population size $N_H = 500$. (**b**) Human population size $N_H = 1000$. Increasing the human population size is equivalent to increase the number of trials in the Bernoulli process and a better agreement is observed for $N_H = 1000$ compared to $N_H = 500$.
