# Supplementary material for: A Unified Framework for the Infection Dynamics of Zoonotic Spillover and Spread
Source: PLoS Negl Trop Dis. 2016 Sep 2;10(9):e0004957. doi: 10.1371/journal.pntd.0004957 (PMC5010258; doi:10.1371/journal.pntd.0004957)
Supplement: S11 Text — Zoonotic Spillover with human-to-human transmission when random effect in the rate are important. (PDF) [file pntd.0004957.s011.pdf]

S11 Text. Application to Lassa Fever. Zoonotic Spillover with human-to-human transmission when random effect in the rate are important.

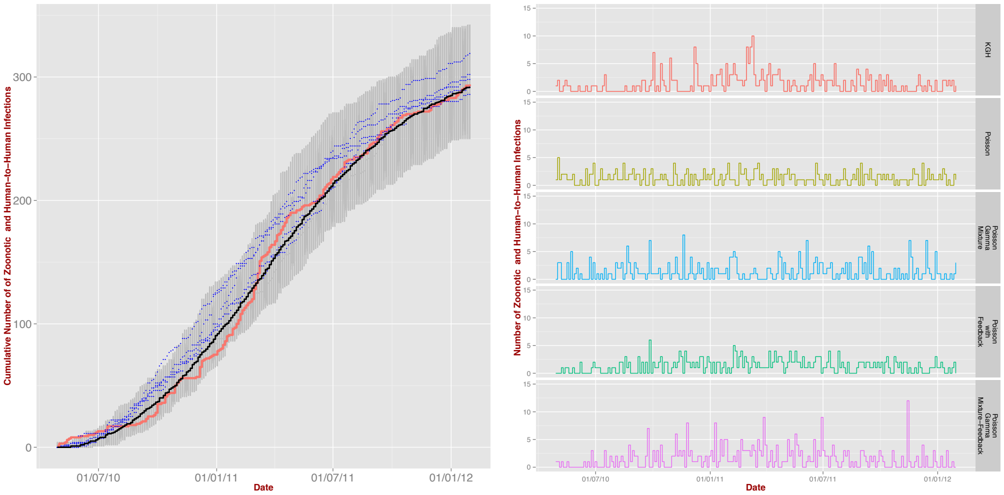

Fig S1. (**a**) Predicted cumulative number of zoonotic and human-to-human infections governed by the 'Poisson-Gamma Mixture with Feedback' model with λ drawn from a gamma distribution (equations (12) and (S2)). (**b**) Time-series of the number of referred/visiting patients at KGH (confirmed cases only) from the 27th of April 2010 to the 31st of January 2012, time resolution $\tau = 3$ days. Four different models are considered: 'Simple Poisson' (Eq (1)); 'Poisson-Gamma Mixture' (Eq (1), with λ drawn from a gamma distribution, Eq (S2)); 'Poisson with Feedback' (equations (5)-(8)); The parameters are optimized with the data from KGH by employing maximum-likelihood ('Simple Poisson' and 'Poisson-Gamma Mixture') and MCMC approach in R [1, 2].

References

1. Park JH, Quinn KM, Martin AD. MCMCpack: Markov Chain Monte Carlo in R. Journal of Statistical Software. 2011;42:323.

2. R Development Core Team. R: A Language and Environment for Statistical Computing; 2011. Available from: `http://www.R-project.org/`.
