# Supplementary material for: A Unified Framework for the Infection Dynamics of Zoonotic Spillover and Spread
Source: PLoS Negl Trop Dis. 2016 Sep 2;10(9):e0004957. doi: 10.1371/journal.pntd.0004957 (PMC5010258; doi:10.1371/journal.pntd.0004957)
Supplement: S12 Text — (PDF) [file pntd.0004957.s012.pdf]

S12 Text. Temporal variations in the parameters. Convex regions in the cumulative number of occurrences and large peaks in the time-series of occurrences can also originate by temporal variations in i) the model parameters and/or ii) the human population size.

Temporal variations in rodent abundance and Lassa virus prevalence were introduced into the model according to the seasonal patterns observed in West Africa for *M. natalensis* captured inside houses and in the proximity of cultivation [1] (Fig S1). In particular, Fig S2.a shows predictions for the cumulative number of spillovers when these are driven by people being exposed solely to infected rodents in houses. This was done by imposing $\eta_H(N_H)\chi_H = 0$ (no human-to-human transmission) and by fitting the overall parameter $Pr_R(N_R)\eta_R(N_R)\chi_R$ with data from Fichet-Calvet *et al.* [1] on trapping success (assumed to be a fraction of total abundance) and Lassa virus prevalence in *M. natalensis* captured inside houses. The patterns of seasonality in the abundance (a proxy for exposure to the reservoir) and disease prevalence cannot explain the initial convex shape in KGH data. Instead, the model predicts an initial concave profile because the parameter $Pr_R(N_R)\eta_R(N_R)\chi_R$ (and thus the rate of spillover) is decreasing during the period from April to January, exactly when the KGH data exhibit the convexity.

Similarly, Fig S2.b shows predictions for the cumulative number of spillovers when these are driven by humans being exposed solely to infected rodents in the proximal cultivation. It is important to note, however, that the data on Lassa virus prevalence in *M. natalensis* captured in these areas are limited to only three time points [1]. Based on these patterns of seasonality for *M. natalensis* abundance and infection prevalence, the model predictions are not able to capture the initial convex shape in the cumulative number of spillovers. Further tests clarified that this is due to the small differences between the maximum and minimum in the parameter $Pr_R(N_R)\eta_R(N_R)\chi_R$.

There are other issues that need further attention. Understanding how such seasonal outcomes interact is of fundamental importance to address a series of questions such as: i) is a continuous exposure more dangerous than an intermittent exposure given the same cumulative viral load? ii) is the risk of spillover higher when the seasonality in reservoir abundance occurs with the same frequency and phase as the seasonality in the pathogen prevalence? There is no obvious answer, especially in the presence of human-to-human transmission. For example, some scenarios might result in a high peak of disease incidence but with long intervals with no disease.

Furthermore, the patterns in the cumulative number of occurrences in the KGH data exhibit a non-linear shape, *i.e.* a convex region for $\sim 6-7$ months followed by a concave region for ~ 12 months. The large extension of these regions suggests that these patterns are not of stochastic origins. In other situations, however, the patterns might not be clear and the statistical significance of these deviations from a corresponding linear profile need to be formally assessed.

Furthermore, the exposure to the reservoir $\eta_R(N_R)$, could also be affected by the size of the human population N_H. For example it is expected that larger human communities have a higher impact on the ecology of the rodents (*M. natalensis* tend to be replaced by *Rattus rattus* and *Mus musculus* in larger human communities and along commercial roads or rails) and thus the exposure η, but this scenario is not included in the current analysis. Patterns of human activity and mobility that affect presence, and thus exposure risk to seasonal *M. natalensis* habitats, would be expected to influence contact rates for different social groups, again, this is beyond the analysis here.

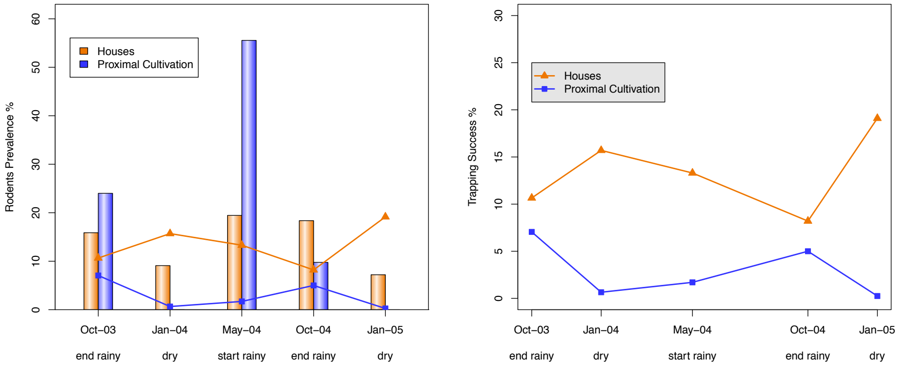

Fig S1. (**a**): Temporal variation of LASV in, and (**b**): mean trapping success in *M. natalensis* by habitat (houses and proximal cultivations), from [1].

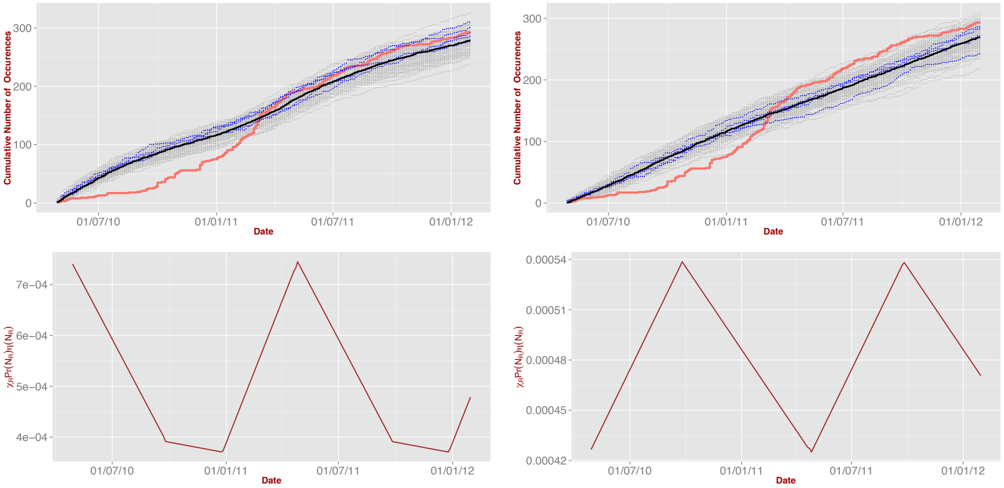

Fig S2. (**a**) Cumulative number of spillovers when these are driven from people being exposed solely to infected rodents in houses. The zoonotic exposure ζ was fitted with a piecewise linear function. Each vertex of the polygonal curve is imposed to be equal to $\alpha Pr_{House} TS_{House}$ where Pr_{House} and TS_{House} are the averaged measured values of LASV prevalence and trapping success [1] at the corresponding time stations for *M. natalensis* captured inside houses; while $\kappa = 0$ (no human-to-human transmission). The value of α is estimated by using MCMC. (**b**) Cumulative number of spillovers when these are driven from people being exposed solely to infected rodents in proximal cultivations. The zoonotic exposure ζ was fitted with a piecewise linear function, each vertex of the polygonal curve is imposed to be equal to $\alpha Pr_{Prox\ Cult} TS_{Prox\ Cult}$ where $Pr_{Prox\ Cult}$ and $TS_{Prox\ Cult}$ are the averaged measured values of LASV prevalence and trapping success [1] at the corresponding time stations for *M. natalensis* captured inside houses; while $\kappa = 0$ (no human-to-human transmission). The value of α is estimated by using MCMC approach.

References

1. Fichet-Calvet E, Lecompte E, Koivogui L, Soropogui B, Doré A, Kourouma F, et al. Fluctuation of abundance and Lassa virus prevalence in Mastomys natalensis in Guinea, West Africa. Vector borne and zoonotic diseases (Larchmont, NY). 2007;7(2):119–28. doi:10.1089/vbz.2006.0520.
