# Supplementary material for: A Unified Framework for the Infection Dynamics of Zoonotic Spillover and Spread
Source: PLoS Negl Trop Dis. 2016 Sep 2;10(9):e0004957. doi: 10.1371/journal.pntd.0004957 (PMC5010258; doi:10.1371/journal.pntd.0004957)
Supplement: S13 Text — (PDF) [file pntd.0004957.s013.pdf]

**S13 Text. Effective Reproductive Number for the simulation generated by the ABM (Zoonotic Spillover with human-to-human transmission).**

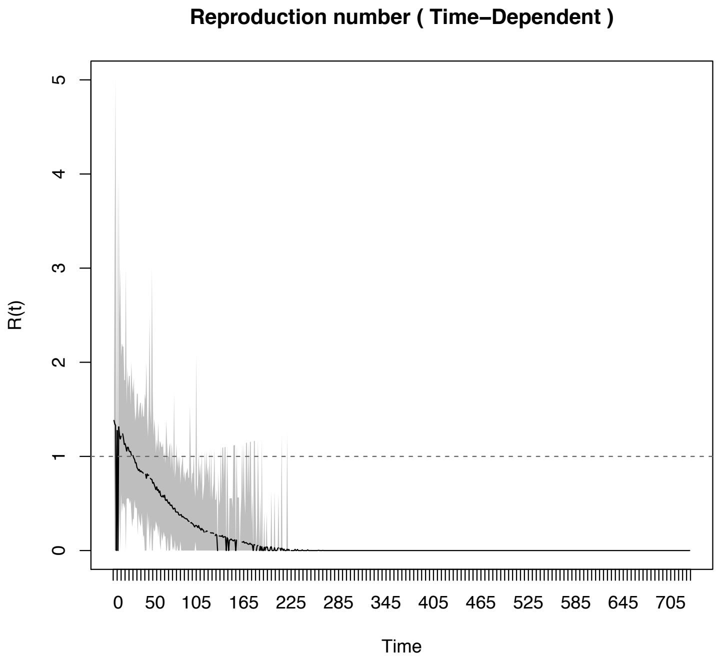


**Fig S1.** Effective Reproductive Number for the simulation generated by the ABM for the zoonotic spillover with human-to-human transmission model ('Poisson with Fedback' model). The Effective Reproductive Number was estimated according to the approach used by [1], with zoonotic spillover events interpreted as imported cases; the distribution of the generation intervals were obtained by the ABM.

# References


1. Wallinga J, Teunis P. Different epidemic curves for severe acute respiratory syndrome reveal similar impacts of control measures. American journal of epidemiology. 2004;160(6):509–16. doi:10.1093/aje/kwh255.
