# Supplementary material for: A Unified Framework for the Infection Dynamics of Zoonotic Spillover and Spread
Source: PLoS Negl Trop Dis. 2016 Sep 2;10(9):e0004957. doi: 10.1371/journal.pntd.0004957 (PMC5010258; doi:10.1371/journal.pntd.0004957)
Supplement: S14 Text — (PDF) [file pntd.0004957.s014.pdf]

S14 Text. Alternative mechanisms leading to a Negative-Binomial. As pointed out by several authors (*e.g.* [1, 2]), the Negative-Binomial distribution might arise from numerous stochastic mechanisms other than the gamma-Poisson mixture mentioned above.

Kendall [3] considered simple birth-death processes in which, each individual would reproduce and die at constant rates, showing that this leads to a Negative-Binomial type of distribution for the population size. This mechanism is not appropriate to describe pure zoonotic processes, as in general, individual spillover events do not 'reproduce' to originate new zoonotic events. This process could be appropriate, however, to describe some classes of human-to-human transmission. In particular, Kendall [3] also studied the situation when, on the top of the birth-death processes described above, there is a chance that a single member will be added to the population by immigration from the outside world with a constant rate. This model, which also lead to a Negative-Binomial distribution, could represent a situation where animal-to-human and human-to-human transmission coexist (like in the Lassa fever case, [4]) under the condition that the birth rate of new infections arising from humans does not depend on the number of susceptibles (*e.g.* when the variation in the number of susceptibles is much smaller compared to the total number of susceptibles).

The Negative-Binomial distribution can be also interpreted as a stuttering-Poisson distribution [5]. This distribution describes a situation when a burst (or cluster) of events are randomly distributed in time (or space) as a Poisson, whilst numbers of events per burst (cluster) are independently distributed as some other distribution. In particular, if the number of bursts (clusters) has a Poisson distribution with mean $-k \ln p$, and the numbers of events are independent random variables logarithmically-distributed as $-\frac{(1-p)^r}{r \ln p}$, then the total number of events per unit time are distributed as a Negative-Binomial shown in Eq (S3) [6]. In the present context, outbreaks of Lassa fever in the reservoir, or peaks in its abundance could be a Poisson distributed burst of events, villages/households can be interpret as spatial clusters. Based on the type of data available, we are not able to ascertain if the Negative-Binomial originates from a stuttering-Poisson distribution and more refined surveillance methods are needed.

We expect, however, that due to the random nature of constituent factors in the rate λ, the Gamma-Poisson mixture is the most appropriate interpretation of the origin of the binomial distribution.

References

1. Kozubowski TJ, Podgorski K. Distributional Properties of the Negative Binomial Lévy Process. Probability and Mathematical Statistics. 2009;29:43–71.

2. Anscombe FJ. Sampling theory of the negative binomial and logarithmic series distributions. Biometrika. 1950;37(3-4):358–82.

3. Kendall DG. Stochastic Processes and Population Growth. Journal of the Royal Statistical Society Series B (Methodological). 1949;11(2):230–282.

4. Lo Iacono G, Cunningham AA, Fichet-Calvet E, Garry RF, Grant DS, Khan SH, et al. Using Modelling to Disentangle the Relative Contributions of Zoonotic and Anthroponotic Transmission: The Case of Lassa Fever. PLoS neglected tropical diseases. 2015;9(1):e3398. doi:10.1371/journal.pntd.0003398.

5. Kemp CD. "Stuttering - Poisson" distributions. Journal of the Statistical and Social Inquiry Society of Ireland. 1967;XX1(V):151–157.

6. Quenouille MH. A relation between the logarithmic, Poisson, and negative binomial series. Biometrics. 1949;5(2):162–4.
